# Coordination of dissolved transition metals in pristine battery electrolyte solutions determined by NMR and EPR spectroscopy†

**DOI:** 10.1039/d4cp01663g

**Published:** 2024-07-09

**Authors:** Jennifer P. Allen, Conrad Szczuka, Holly E. Smith, Erlendur Jónsson, Rüdiger-A. Eichel, Josef Granwehr, Clare P. Grey

**Affiliations:** a Yusuf Hamied Department of Chemistry, University of Cambridge Lensfield Road Cambridge CB2 1EW Cambridge UK cpg27@cam.ac.uk; b The Faraday Institution, Quad One, Harwell Science and Innovation Campus Didcot OX11 0RA UK; c Institute of Energy and Climate Research (IEK-9), Forschungszentrum Jülich GmbH 52425 Jülich Germany; d Institute of Physical Chemistry, RWTH Aachen University 52056 Aachen Germany; e Institute of Technical and Macromolecular Chemistry, RWTH Aachen University 52056 Aachen Germany

## Abstract

The solvation of dissolved transition metal ions in lithium-ion battery electrolytes is not well-characterised experimentally, although it is important for battery degradation mechanisms governed by metal dissolution, deposition, and reactivity in solution. This work identifies the coordinating species in the Mn^2+^ and Ni^2+^ solvation spheres in LiPF_6_/LiTFSI–carbonate electrolyte solutions by examining the electron–nuclear spin interactions, which are probed by pulsed EPR and paramagnetic NMR spectroscopy. These techniques investigate solvation in frozen electrolytes and in the liquid state at ambient temperature, respectively, also probing the bound states and dynamics of the complexes involving the ions. Mn^2+^ and Ni^2+^ are shown to primarily coordinate to ethylene carbonate (EC) in the first coordination sphere, while PF_6_^−^ is found primarily in the second coordination sphere, although a degree of contact ion pairing does appear to occur, particularly in electrolytes with low EC concentrations. NMR results suggest that Mn^2+^ coordinates more strongly to PF_6_^−^ than to TFSI^−^, while the opposite is true for Ni^2+^. This work provides a framework to experimentally determine the coordination spheres of paramagnetic metals in battery electrolyte solutions.

## Abstract 2

An NMR and EPR study of the solvation shells of typical transition metal ions dissolved from lithium-ion battery cathode materials.

## Graphical abstract

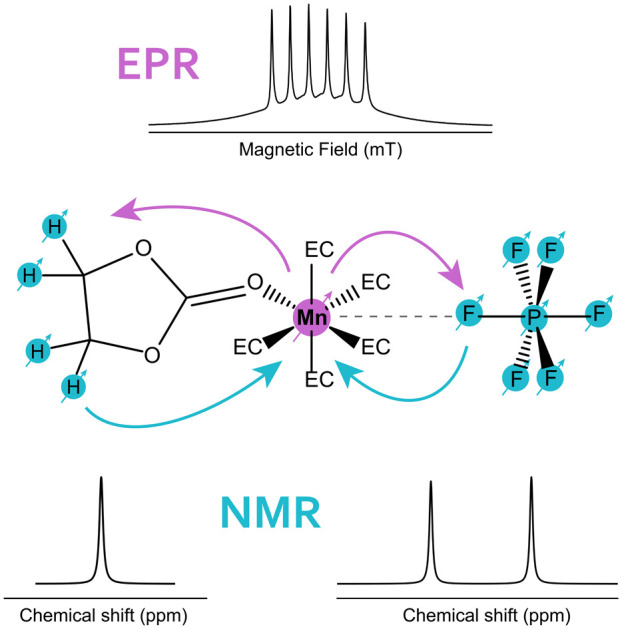

## Introduction

As lithium-ion cells degrade, transition metal ions may dissolve from cathode materials and be deposited at the anode, causing issues including degradation of the solid electrolyte interphase (SEI) at the anode, further SEI formation and increased trapping of active lithium, impedance rise, and capacity loss.^1–5^ While many studies have been conducted on the topic of Li^+^ solvation in battery electrolytes, there is far less work surrounding the solvation of dissolved transition metals. Yet an understanding of the nature and strength of coordination of the transition metal ions by the different species in both pristine (fresh) and degraded electrolyte solutions is important to understand and control both the dissolution of transition metals from the cathode and their deposition on the anode, ultimately providing chemical insights into cell degradation pathways.

The picture of Li^+^ solvation in battery electrolyte solutions is itself complicated; however, it is understood that Li^+^ tends to be tetrahedrally solvated^6–10^ and that coordination to ethylene carbonate (EC) is preferred over coordination to linear carbonates,^7,9–17^ with the extent of ion pairing between Li^+^ and PF_6_^−^ being dependent on the salt concentration.^11,18,19^ For transition metal solvation, a computational study of Mn^2+^, Ni^2+^, and Co^2+^ solvation by EC showed that the structures would likely be six-coordinate, and that Mn^2+^ desolvation occurs more readily than Ni^2+^ or Co^2+^ desolvation, potentially influencing the role of Mn^2+^ in the SEI.^20^ Later simulations of the Mn^2+^ solvation shell showed that Mn^2+^ interaction energies follow the order EC > PF_6_^−^ > linear carbonates.^21^ Our previous NMR studies have suggested that dissolved transition metals may favour coordination to EC over ethyl methyl carbonate^22,23^ and may coordinate preferentially to PF_6_^−^ degradation products over pristine electrolyte components.^23,24^

In addition to understanding the solvation of dissolved transition metal ions, we aim to understand the solvation of model transition metal salts, and identify whether these model species are truly representative. Most studies aiming to mimic the effects of dissolved metals from cathode materials, including this work, use commercially available M(TFSI)_2_ salts.^21,25–34^ Although it is assumed that the metal cations dissociate from the TFSI^−^ anions, (*i.e.*, tight M^2+^–TFSI^−^ ion pairs are not formed) due to the high solubility of the metal salts, and their small concentrations within the electrolyte, the extent of coordination between dissolved metals and TFSI^−^ is not yet clear. At these low concentrations, the TFSI^−^ anion is not thought to alter the reduction potential of dissolved transition metals or to affect cell cycling.^28^ It has also been suggested that because electrolyte solutions containing LiTFSI and LiPF_6_ behave similarly with respect to gassing at the negative electrode,^35–37^ the TFSI^−^ counterion that is added with transition metals to an LiPF_6_ solution should not affect the electrolyte decomposition reactions induced by those transition metals.^26^ However, a comparison of electrolyte solutions containing added Mn(TFSI)_2_ and Mn(PF_6_)_2_ produced different cycling behaviour in LiFePO_4_/graphite cells, even though cells were not negatively affected by the addition of LiTFSI.^29^ Specifically, differences were observed in the amount of Mn deposited at both electrode surfaces and in the rate of capacity loss, with rapid initial capacity loss caused by Mn(TFSI)_2_ and smaller, continual capacity loss with more ongoing parasitic side reactions caused by Mn(PF_6_)_2_.^29^ A potential explanation for this is that Mn(TFSI)_2_ may not dissociate completely in a typical electrolyte solution comprising LiPF_6_, EC, and linear carbonates; such a notion is consistent with calculations showing that the Mn^2+^ interaction energy with TFSI^−^ is even stronger than with EC, PF_6_^−^, or linear carbonates.^21^ In a similar vein, a study of Co deposition using the additive Co(NO_3_)_2_ found deposition of Li_3_N, which was not present when LiNO_3_ was added to the electrolyte solution.^38^ We note that the SEIs formed in electrolytes comprising LiTFSI are very different from those formed with LiPF_6_, which may also play an indirect role in some of the observed experimental differences. However, if the compounds that are used to mimic the effects of transition metal dissolution are found to alter the transition metal coordination shells, such that the counterions affect the action of dissolved transition metals, then studies using transition metal salts may be non-representative and lead to flawed conclusions about the role of dissolved transition metal ions in lithium-ion cells.

Transition metals are largely believed to dissolve from cathode materials in the +2 oxidation state (*e.g.*, Ni^2+^, Mn^2+^, Co^2+^).^5,22,28,39–42^ These species are paramagnetic, enabling the use of paramagnetic NMR spectroscopy and, in some cases, EPR spectroscopy. In NMR, the nuclei that are located near paramagnetic species undergo rapid relaxation, permitting an indirect understanding of the coordination sphere of the paramagnetic centre; in pulsed EPR, the paramagnetic centre is probed directly and the surrounding magnetic nuclei affect the resonance conditions. In EPR spectroscopy, transition metal ions are often difficult to study if they have integer spins, as is the case for the d^8^*S* = 1 Ni^2+^ ion, because of the zero field splittings and short relaxation times. In contrast, dilute high-spin d^5^ Mn^2+^ ions are well-suited for EPR studies, because the half-filled outer d shell ensures a small-to-zero ground-state orbital angular momentum, especially for cubic symmetry,^43^ and small zero field splittings for the central transitions of the *S* = 5/2 spin system. Consequently, anisotropies are small and electronic relaxation times are comparably long, since modulations in the spin–orbit couplings no longer drive significant relaxation effects.^44^ Provided electronic relaxation times *T*_1,2e_ are sufficiently long, pulsed EPR techniques probing small electron–nuclear (hyperfine) interactions are particularly useful for ligand identification. These specialised techniques are necessary since inhomogeneous EPR line broadening typically exceeds the resolution limit necessary to identify hyperfine couplings in transition metal ion complexes apart from those involving the central nucleus. Measurements are performed on frozen solutions at cryogenic temperatures to ensure sufficiently long *T*_1,2e_ as well as rigid and potentially well-defined complexes. Although dynamics, as probed by NMR spectroscopy, are inaccessible as a consequence, an ensemble of structural snapshots should be generated during freezing, which should capture the thermodynamic ground state.

We have previously shown that the presence of dissolved paramagnetic ions can result in differential enhancement of the NMR relaxation rates of the signals from a variety of species in both pristine and degraded battery electrolyte solutions.^23,24^ We have used these relaxation rates, coupled with the accompanying bulk magnetic susceptibility shifts, to quantify metal concentrations and investigate binding to different electrolyte solvents. We have also used EPR to, for example, establish the solvation shell of dissolved vanadyl ions.^45,46^ Here, we combine the direct observation of the paramagnetic ions by EPR with solution NMR measurements to characterise the solvation shells of both Mn^2+^ and Ni^2+^ in pristine battery electrolytes. Model Mn(TFSI)_2_ and Ni(TFSI)_2_ solutions with varying concentrations of EC, LiPF_6_, and LiTFSI are investigated.

Static Mn^2+^ complexes are examined at low temperature in frozen solutions *via* EPR, field-swept echo and electron nuclear double resonance (ENDOR) experiments, revealing their complex symmetry and the magnetic nuclei in surrounding molecules. In liquids, at ambient temperatures, Mn^2+^ and Ni^2+^ solvation is studied dynamically *via*^1^H and ^19^F longitudinal and transverse nuclear relaxation rates, and the favourability of coordination to EC *vs.* PF_6_^−^ in pristine electrolyte solutions is probed. Interactions with PF_6_^−^*vs.* TFSI^−^ are then explored to determine whether M(TFSI)_2_ salts, added to mimic the effects of transition metal dissolution, alter the transition metal coordination shells. While transition metals are unlikely to accumulate in the electrolyte solution of a full cell undergoing charge–discharge cycling in the concentrations used in this work (1–8 mM *vs.*, for example, the ∼0.1 mM Ni concentrations found in electrolytes from cycled LiNi_0.8_Mn_0.1_Co_0.1_O_2_ cells),^47,48^ we consider the concentrations used in this work to be small enough to be representative of the solvation shell of metals dissolved at very low concentrations. That is, the metals are present in low enough concentrations that the pristine electrolyte components are still in significant excess, and no metal clustering effects or other high-concentration effects should occur. We also note that soaking of cathode materials in battery electrolytes, often used to assess dissolution, can cause Mn or Ni accumulation in the electrolyte beyond what is typically observed in cycling cells—particularly in high-temperature soaking experiments, for example, with LiMn_2_O_4_ or LiNi_0.5_Mn_1.5_O_4_.^42,49–51^ While this work centres on coordination in pristine lithium-ion battery electrolytes, the approaches developed and employed herein are suitable to probe Mn^2+^ dissolution in any electrolyte solution or cell chemistry.

## Methods

### Electrolyte solutions

Transition metal bis(trifluoromethane)sulfonimide (TFSI) salts were added to electrolytes to mimic dissolved transition metals: Mn(TFSI)_2_ (Solvionic, 99.5%) and Ni(TFSI)_2_ (Alfa Aesar, ≥97%). Ethylene carbonate (EC) and ethyl methyl carbonate (EMC) were used as electrolyte solvents. Solutions used in this work include: 1 M LiPF_6_ in 3 : 7 EC : EMC (for EPR and NMR); 1 M LiTFSI in 3 : 7 EC : EMC (for EPR and NMR); 1 M LiPF_6_ in EMC (for NMR); and 3 : 7 EC : EMC (for NMR). All solvent ratios are given as volume ratios (v/v). Solutions were prepared in an argon glovebox. For EPR experiments: Solutions were mixed in-house, using LiPF_6_ (Sigma Aldrich, ≥99.99% trace metals basis), LiTFSI (Alfa Aesar, 98+%), EC (Sigma Aldrich, 99%, anhydrous), and EMC (Sigma Aldrich, 99%). Mn(TFSI)_2_ was dried at 110 °C under dynamic vacuum for three days. For NMR experiments: 1 M LiPF_6_ in 3 : 7 EC : EMC was sourced premixed (soulbrain MI PuriEL R&D 280); 3 : 7 EC : EMC was sourced premixed (soulbrain and Solvionic); 1 M LiTFSI in 3 : 7 EC : EMC was mixed in-house (99.95% LiTFSI, Sigma Aldrich, with premixed EC : EMC); and 1 M LiPF_6_ in EMC was mixed in-house (99.99% LiPF_6_, Solvionic; 99.9% EMC, Solvionic). Salts were dried at 100 °C under vacuum before use.

### EPR spectroscopy

Samples for EPR experiments were freshly prepared by dissolving 8 mM of Mn(TFSI)_2_ in the electrolyte stock solution. The obtained solution was transferred into a 2 mm outer diameter EPR tube (Wilmad, CFQ) for X-band and into a 0.9 mm outer diameter tube (Wilmad, Suprasil) for Q-band experiments. Tubes were sealed and transferred from the glovebox into the pre-cooled EPR resonator within 15 min after preparation.

EPR experiments were conducted on a Bruker ElexSys E580 spectrometer at a temperature of 20 K, maintained within a helium cryostat (Oxford Instruments, CF935). The EPR resonator was pre-cooled before inserting the sample, to rapidly cool (flash-freeze) the sample. (It should be noted that EPR/ENDOR experiments performed by more slowly cooling the resonator over approximately 15 minutes did not yield observable differences.)

Microwave pulses were amplified using a 1 kW travelling-wave tube amplifier, radiofrequency pulses with a 150 W amplifier. Measurements were performed using an EN4118X-MD4 resonator for X-band and an EN5107-D2 resonator for Q-band microwave frequencies. Field-swept pulsed EPR spectra were obtained by integration in a window of 62 ns centred at the Hahn echo. For X-band, *τ* = 180 ns and the pulse durations were *t*_π/2_ = 12 ns and *t*_π_ = 24 ns. For Q-band, *τ* = 160 ns and pulse durations were *t*_π/2_ = 20 ns and *t*_π_ = 40 ns. A two-step phase cycle was applied. Davies-type electron nuclear double resonance (ENDOR) experiments were conducted at Q-band using the pulse sequence π–*D*–π/2–*τ*–π–*τ*-echo for the microwaves with *t*_π/2_ = 100 ns, *t*_π_ = 200 ns, and *τ* = 450 ns. During the delay *D*, radiofrequency π-pulses (at close to the ^1^H Larmor frequency) lasting 10 μs were applied. ENDOR experiments were acquired at a frequency offset that corresponds to the maximum of the low-field signal, corresponding to the ^55^Mn nuclear spin manifold *m*_*I*_ = −5/2.

### EPR spectra simulation

EPR spectra simulations were performed by using *EasySpin v6.0.0-dev.49*^52,53^ running in *Matlab* v2020b (MathWorks). ^1^H powder Davies ENDOR spectra were simulated taking into account all specified nuclei with hyperfine tensor components as obtained from DFT calculations. *Easyspin*'s simulation module salt was used, applying second-order perturbation theory and the product rule. The obtained spectra were multiplied with the Davies ENDOR detection function^54,55^1
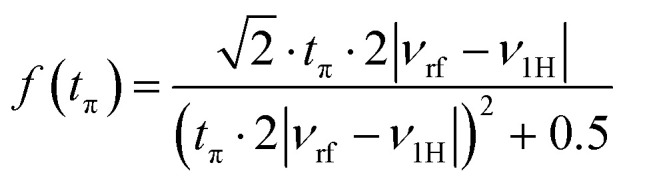
with the pulse duration *t*_π_ = 0.2 μs of a selective π pulse, the applied RF frequency *ν*_rf_ and the ^1^H Larmor-frequency *ν*_1H_. Ultimately, a Gaussian convolution (FWHM = 0.1 MHz) was applied.

Field-swept echo-detected spectra were fitted using the *EasySpin* fitting module pepper. The fit was initialised using an electronic spin 5/2 exhibiting an isotropic *g* tensor and an anisotropic zero-field splitting, coupled to a ^55^Mn nucleus with isotropic hyperfine coupling. A convolutional Gaussian with a full width at half maximum (FWHM) of 6 MHz for X-band and 13 MHz for Q-band was used along with anisotropic Gaussian zero-field splitting parameter strain.

### NMR spectroscopy

For variable temperature NMR, ^1^H and ^19^F NMR relaxation times were measured on a Bruker Avance III HD 500 MHz spectrometer using a broadband observe (BBO) probe. A sealed capillary of C_6_D_6_ was used for field locking. Longitudinal, *T*_1_, relaxation times were measured using the inversion recovery pulse sequence and transverse, *T*_2_, relaxation times were measured using the Carr–Purcell–Meiboom–Gill (CPMG) pulse sequence^56,57^ with 2 ms echo spacings. Experiments were performed at 0, 15, 30, 45, and 60 °C.

Ambient temperature relaxation experiments were performed on a Bruker Avance III HD 300 MHz spectrometer equipped with a Bruker double-channel MicWB40 probe. No deuterated solvents were incorporated into the electrolyte samples. *T*_1_ values were measured using inversion recovery and *T*_2_ values were measured using CPMG, with 2 ms echo spacings (*τ*) for diamagnetic and Ni^2+^-containing solutions. Due to fast relaxation, for Mn^2+^-containing solutions *τ* = 0.05–2 ms was used. Use of shorter *τ* values did not significantly impact the *T*_2_ measurements. For all NMR experiments, J-Young NMR tubes were filled and sealed in an argon glovebox.

### Viscosity measurements

Kinematic viscosities of EMC, 3 : 7 EC : EMC (v/v), 1 M LiPF_6_ in EMC, 1 M LiPF_6_ in 3 : 7 EC : EMC, and 1 M LiTFSI in 3 : 7 EC : EMC were measured in an argon glovebox with a Micro-Ostwald viscometer, type 51610/1 (Xylem Analytics), with an instrument constant *K* = 0.01063 mm^2^ s^−2^. A volume of 2 mL of each solution was used for viscosity measurements. Temperature varied from 25.7–27.2 °C. Kinematic viscosities were converted to dynamic viscosities by multiplying by solution density; solution density was measured by weighing 1 mL of solution in an argon glovebox.

### DFT calculations

The density functional theory (DFT) calculations used the software package *ORCA, version 5.0.1*.^58^ Geometry optimisation was performed on [Mn(EC)_4_]^2+^, [Mn(EC)_5_PF_6_]^2+^, and [Mn(EC)_6_]^2+^ (identified as being the likeliest candidate in a set of DFT calculations described in the ESI†), with the TPSSh^59,60^ hybrid functional, approximating the relativistic Hamiltonian with the zeroth-order regular approximation (ZORA).^61^ Def2-TZVP(-f) basis sets^62^ in their ZORA-recontracted version^63^ were used. Decontracted def2/J auxiliary basis sets^64^ were employed for the resolution-of-identity and chain-of-spheres approximation.^65^ Convergence was attained by a tight self-consistent-field criterion. Hyperfine coupling tensors were calculated with the hybrid functional TPSSh and the ZORA relativistic approximation, as described recently.^45,46^ ZORA-def2-TZVP(-f) basis sets were modified through full decontraction of s shells and adding three more Gaussians, with exponents calculated by multiplying the steepest original primitive with 2.5, 6.25, and 15.625.^66^ Spin–orbit coupling was taken into account with the spin–orbit mean-field approximation (SOMF).^67^ To increase integration accuracy, the defgrid3 setting was chosen with radial accuracy IntAcc further increased to 11 for manganese and 9 for all other atoms.

## NMR relaxation theory

In this work, Solomon–Bloembergen–Morgan (SBM) theory is applied to interpret measured NMR relaxation times.^68–72^ Within this theory, relaxation is considered to be driven by a dipolar term (the Solomon equations)^70^ and a contact term (the Bloembergen equations).^71^ The dipolar term treats the through-space coupling of the nucleus and unpaired electron as an interaction between two point dipoles, *i.e.*, the unpaired electron is localised on the paramagnetic ion, while the isotropic Fermi contact term results from the unpaired electron density present at the nucleus.^68,69^ The relaxation of a nucleus bound or close to a paramagnetic metal centre, M, is described by2
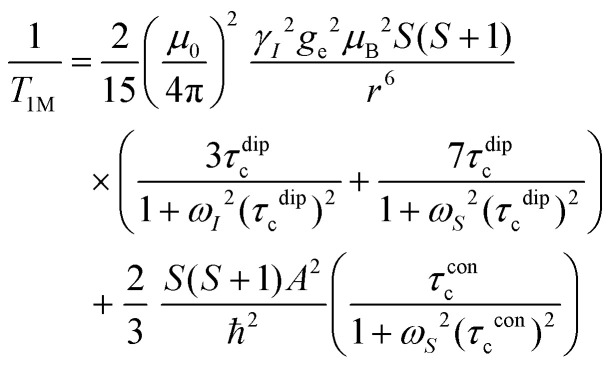
3
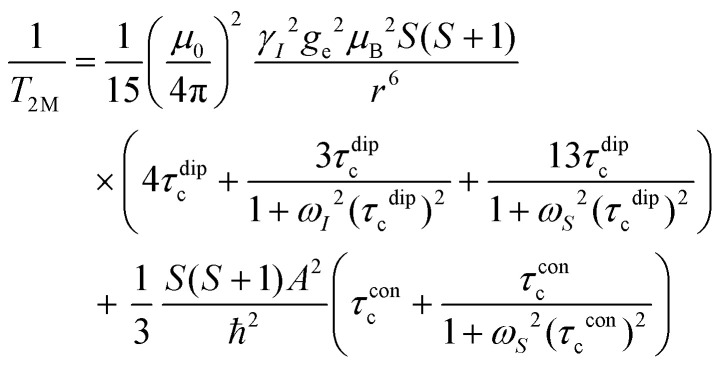
where *T*_1M_ and *T*_2M_ indicate the paramagnetic contributions to the longitudinal and transverse relaxation times and 1/*T*_1M_ = *R*_1M_ and 1/*T*_2M_ = *R*_2M_ the relaxation rates. Other terms are the permeability of a vacuum *μ*_0_; nuclear gyromagnetic ratio *γ*_*I*_; electron spin *g*-factor *g*_e_; Bohr magneton *μ*_B_; electron spin *S*; distance between the nucleus and paramagnetic ion *r*; correlation time associated with the dipolar interactions *τ*^dip^_c_; Larmor frequencies for the nuclear spin, *ω*_*I*_, and the electron spin, *ω*_*S*_; contact term of the hyperfine interaction constant *A* and reduced Planck constant *ħ* (*A*/*ħ* in rad s^−1^, or *A*/*h* in Hz); and correlation time for the contact term *τ*^con^_c_.

Molecular rotation results in fluctuations arising from the orientation dependence of the anisotropic dipolar coupling tensor, thus the correlation time for the dipolar term

4 (*τ*^dip^_c_)^−1^ = *τ*_r_^−1^ + *τ*_e_^−1^ + *τ*_M_^−1^

incorporates the correlation time for molecular rotation *τ*_r_, the electronic relaxation time *τ*_e_, and the lifetime of the inner sphere complex *τ*_M_, which can also be quantified *via* the chemical exchange time. The rotational correlation time can be approximated from the viscosity *η*, temperature *T*, Boltzmann constant *k*_B_, and the molecular volume, assuming rigid spherical particles, using the Stokes–Einstein equation5
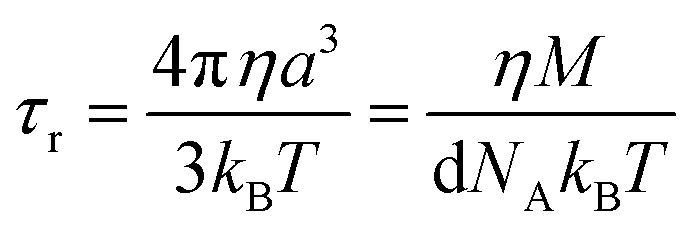
where *a* is the molecular radius, *M* is the molar mass, *d* is the density, and *N*_A_ is Avogadro's number. Since the contact term is isotropic, its correlation time *τ*^con^_c_ incorporates *τ*_e_ and *τ*_M_ only.

6 (*τ*^con^_c_)^−1^ = *τ*_e_^−1^ + *τ*_M_^−1^

Within SBM theory, the correlation times *τ*^dip^_c_ or *τ*^con^_c_ are generally dominated by whichever is shortest of the contributing correlation times in eqn (4) and (6). For small molecules, like those in non-viscous battery electrolyte solutions, *τ*_r_ is short (∼10^−10^ s),^68^ so *τ*^dip^_c_ cannot be much longer than ∼10^−10^ s, even if *τ*_e_ and *τ*_M_ are very long. However, since *τ*^con^_c_ is not governed by molecular rotation, and in cases where the electronic relaxation and chemical exchange are very slow, *τ*^con^_c_ can be very long, so that *τ*^con^_c_ ≫ *τ*^dip^_c_. Unlike *R*_1M_, the expression for *R*_2M_ contains terms that are linear in *τ*^con^_c_ and thus *R*_2M_ may become significantly larger than *R*_1M_ in this regime.^71,73^ The contact term also becomes more important when the nucleus being probed is closer to the coordination site in an inner sphere complex, as this increases the hyperfine interaction.^74^

At the magnetic fields used in this work, the Larmor frequency of the electron spin, *ω*_*S*_, is very large.^71,75,76^ Since Mn^2+^ ions undergo relatively slow electronic relaxation, and thus *τ*_e_ is long,^68^ all the terms involving 1/*ω*_*S*_^2^ are expected to be small and can be ignored to a first approximation.^71,75,77^ For Mn^2+^, eqn (2) and (3) can therefore be approximated as7
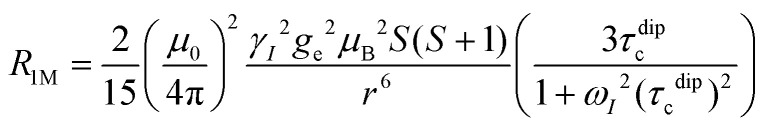
8
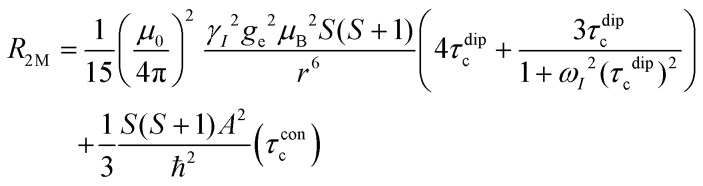
Notably, the simplified expression for *R*_1M_ is reduced to only a dipolar term, while the expression for *R*_2M_ retains dipolar and contact terms.

In most solutions, NMR nuclei are not statically bound to a paramagnetic centre. The measured relaxation rates *R*_1_ and *R*_2_ depend on the timescale and nature of the exchange between bound and unbound species, and whether inner or outer sphere complexes are formed.^68,74^ If we consider a system with rapid exchange between bound and unbound ligands, assuming only an inner sphere relaxation mechanism, then the fast-exchange limit of paramagnetic contribution to the relaxation time is given by *R*_1p_ = *f*_M_*R*_1M_ and *R*_2p_ = *f*_M_*R*_2M_, where *f*_M_ indicates the molar fraction of the species being probed that is coordinated to the transition metal. For intermediate exchange rates, *τ*_M_^−1^, more complicated expressions can be derived. For *R*_1_, the terms are relatively simple, where *R*_1d_ is the diamagnetic longitudinal relaxation rate,9
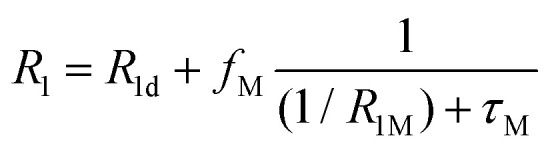
Expressions for *R*_2_ are more complicated because they also depend on the hyperfine shift, Δ*ω*_M_, for the bound species. An intermediate regime exists when *τ*_M_^−1^ is of the same order of magnitude as Δ*ω*_M_, and additional linebroadening is observed,10
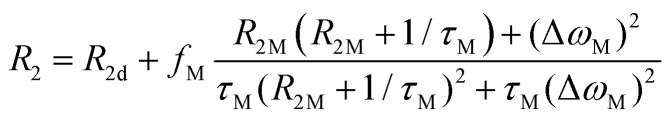
where *R*_2d_ is the diamagnetic transverse relaxation rate. As *τ*_M_^−1^ approaches zero, *R*_1p_ and *R*_2p_ both approach zero for an inner sphere complex. The *f*_M_ term accounts for the transition metal concentration, the concentration of the solution species being probed, and the solvation number of the solution species in the transition metal coordination sphere. Notably, the paramagnetic relaxation components *R*_1p_ = *R*_1_ − *R*_1d_ and *R*_2p_ = *R*_2_ − *R*_2d_ are directly proportional to *f*_M_. The expressions are further complicated in a multicomponent electrolyte solution, where we must also account for the exchange of several different species in and out of the coordination shell. Lastly, we again note that these equations represent paramagnetic relaxation as entirely arising from inner sphere coordination. Relaxation in the second coordination sphere should be dominated by the dipolar term, but it may depend on both a diffusional correlation time, *τ*_D_, and *τ*_e_.^68^ Correlation times may also vary between inner sphere, outer sphere, and bulk species. Additional chemical shift variations may arise for ^19^F of PF_6_^−^ in the second coordination sphere, which may break the symmetry of the ion and lead to additional *R*_2_ effects. Since the SBM theory was derived for binary mixtures, an analysis of the investigated electrolyte systems is not expected to be quantitative, yet it provides a robust framework for qualitative conclusions.

## Results and discussion

### EPR spectroscopy, 20 K

#### Echo-detected EPR spectra

**Fig. 1:**
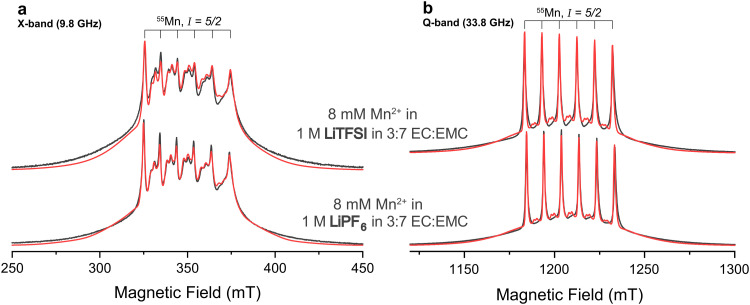
(a) X-band and (b) Q-band experimental pulsed EPR spectra recorded using field-swept Hahn-echoes (black) and spin Hamiltonian fits (red). Mn^2+^ is studied in premixed electrolytes with either LiPF_6_ (bottom) or LiTFSI (top) salts. All fits used identical interaction parameters but different line broadening (Table 1).

Frozen solutions of 8 mM Mn(TFSI)_2_ dissolved in premixed electrolytes containing 1 M LiPF_6_ or LiTFSI in a volumetric 3 : 7 ratio of EC and EMC were studied at a temperature of 20 K. Fig. 1 shows field-swept echo-detected pulsed EPR spectra of the two samples at X- and Q-band. The spectra are dominated by the |−1/2〉 ↔ |1/2〉 transitions of the high-spin d^5^ complex. The six-fold line splitting, clearly evident at Q-band, indicates strong hyperfine coupling to the ^55^Mn nucleus of similar magnitude for both samples. The sets of doublets in between the central transitions are formally forbidden transitions originating from zero-field splitting interactions intermixing *m*_*I*_ states,^78^ indicating deviation from perfect cubic symmetry. Their presence is more evident at X-band since the zero-field splitting is less effectively quenched by the electron Zeeman effect at this lower static magnetic field. The observation of well-resolved Mn-hyperfine splittings is clear evidence for the Mn-ions being diluted in the EC/EMC/LiTFSI/LiPF_6_ frozen matrix, consistent with the low concentration of this ion.^79^ Close Mn–Mn proximity in, for example, a precipitated Mn salt would result in exchange interactions and more broadening/disappearance of the hyperfine splittings. The broad featureless outer transitions originate from electron-spin transitions other than the |−1/2〉 ↔ |1/2〉 transitions. The two samples appear to possess very similar electronic interaction parameters, but Mn^2+^ in the LiTFSI electrolyte exhibits a larger line broadening, evident from the reduced relative intensity of the sextet (Fig. S1, ESI†).

#### Magnetic interaction parameters

The pulsed EPR spectra were modelled and fitted (Fig. 1, red traces) with a spin Hamiltonian that incorporates electronic and nuclear spin operators and rank 2 interaction tensors. Values for *g* and *A*(^55^Mn) could be extracted more accurately *via* the Q-band rather than the X-band spectra. Identical interaction parameters and symmetries were obtained for both samples (Table 1 and Fig. S1, ESI†), indicating that the electrolyte salt anion, PF_6_^−^ or TFSI^−^, has little effect on the magnetic environment of the Mn^2+^ ion. The electronic *g* tensor is set to be isotropic because no anisotropy was resolved and the observed minor deviation from the free electron value (Δ*g* = 0.0007) is common for high-spin d^5^ complexes. Similarly, the hyperfine coupling to the central atom *A*(^55^Mn) is determined to be isotropic, indicating that it largely arises from the Fermi-contact interaction caused by spin polarisation of s shells. The sign of *A*(^55^Mn) cannot be directly inferred from the data but is typically negative for Mn^2+^ complexes.^80^

**Table 1:** Extracted electronic parameters from EPR relaxation measurements at 20 K and spectral fitting using the spin Hamiltonian formalism. Fit errors are derived from least-squares fits. Systematic errors might exceed the given uncertainties

	Mn^2+^ in 1 M LiPF_6_ in 3 : 7 EC : EMC	Mn^2+^ in 1 M LiTFSI in 3 : 7 EC : EMC
From spin Hamiltonian fit		
Isotropic *g*-value	2.0016 ± 0.0002	
|*A*|(^55^Mn)| (MHz)	273 ± 5	
|*D*| (MHz)	415 ± 60	
|*E*/*D*| (MHz)	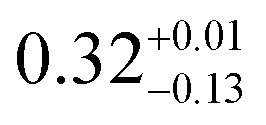	
*D*, *E* strain (MHz)	230, 60	330, 120

From relaxation measurements^a^		
*T*_2e_ (μs)	0.37	0.49
*β*_*T*_2e__	1.26	1.29
*T*_1e_ (μs)	6.16	8.65
*β*_*T*_1e__	0.79	0.77

a Fitted with a stretched/compressed exponential: *S*(*t*) = exp(−(*t*/*T*_e_)^*β*^) + *y*_0_.

Further extractable parameters describe the zero-field splitting arising from the interaction of multiple unpaired electrons within the same (d^5^) ion, which can be described by the electron-spin Hamiltonian term

11 *S⃑***D***S⃑* = *D*(*Ŝ*_*z*_^2^ − *Ŝ*^2^/3) + *E*(*Ŝ*_*x*_^2^ − *Ŝ*_*y*_^2^)

where the tensor **D** is expressed by the scalar parameters *D* and *E*, representing the axial/tetragonal and rhombic distortion, respectively, with 0 < |*E*/*D*| < 0.33. These parameters are affected by the symmetry and type of ligands. A least-squares fit of the experimental spectrum was performed with |*D*| ≈ 415 MHz, where the intensity and position of the forbidden central transitions are dominant in influencing the fit result. Additional simulations with the aim of reproducing the outer transitions reveal that |*D*| might be larger, and approximately 500–600 MHz (Fig. S2, ESI†). Imperfect fitting can result from a superposition of several similar ligand spheres/conformers of Mn^2+^ complexes or from field-dependent relaxation time dispersion. Conclusively, however, |*D*| is determined to be on the order of several hundred MHz. Furthermore, |*E*/*D*| = 0.32 was extracted from the least-squares fit. Despite considerable uncertainty, large rhombicity is indicated by intensity ratios and positions of forbidden transitions in the regions of low- and high-field central transitions, which are sensitive to |*E*/*D*| (Fig. S3, ESI†).

Predominantly, Mn^2+^ complexes are sixfold coordinated in solution, while occasionally five- or sevenfold, and fourfold coordination can occur if halogens and oxygen are directly coordinated.^81,82^ For sixfold coordinated Mn^2+^ with close to octahedral symmetry, *D* is often comparably small, on the order of |*D*| ≈ 300 MHz or smaller.^43^ The experimental value extracted here falls in the upper limit of that range, but is at least an order of magnitude smaller than values for known fivefold oxygen coordination or fourfold halogen/oxygen coordination.^81^ The exact value of *D* will depend on the orientation of the ligands,^83^*i.e.*, degrees of freedom along dihedral angles, the composition and freezing behaviour of the solvent mixture,^78^ and the exact geometry, particularly the bond length between the transition metal and the first ligand atom.^84^ Furthermore, the experimentally determined zero-field splitting tensor exhibits large rhombicity. This rhombicity may also result from different participating ligands in the first coordination sphere. Assuming symmetric sixfold carbonate coordination of Mn^2+^*via* oxygen following the arguments above, then solvent-separated anions surrounding the complex may also be responsible for the rhombicity. One charge-compensating anion situated around the central atom would favour axial symmetry, as would two with anion–Mn^2+^–anion angles of 180°; two anions with 90° angles would favour rhombic symmetry. The only extractable difference from the pulsed EPR spectrum between the two samples under investigation is the estimated breadth of *D* and *E* distribution, given as strain parameters. These are significantly higher for the sample involving TFSI^−^ anions. Again, assuming a solvent-separated ion pair, the *O*_h_ symmetry of the PF_6_^−^ anion may result in a more ordered inner and outer coordination sphere compared to the TFSI^−^ anion with lower symmetry and a flatter total energy landscape. The coordination of the anions is explored further below *via* double resonance EPR experiments.

#### Electronic relaxation

Electronic relaxation times were estimated using the Hahn-echo and inversion recovery pulse sequences for the spin–spin (*T*_2e_) and spin–lattice (*T*_1e_) relaxation times, respectively. Fitting was performed with a stretched/compressed exponential function, the Hahn-echo traces exhibiting a compressed exponential behaviour with a stretching exponent *β*_*T*_2e__ > 1, characteristic for Gaussian relaxation often caused by dipole–dipole interactions. However, this is also characteristic for pulsed EPR when a limited excitation bandwidth triggers apparent relaxation effects such as spectral and instantaneous diffusion.^85^ In contrast, the inversion recovery traces exhibit a stretched exponential behaviour with *β*_*T*_1e__ < 1, indicating a distribution of relaxation times, which could be caused by a distribution of conformers or varying ligand combinations. For the sample containing TFSI^−^, the *T*_1e_ and *T*_2e_ values are 40% and 30% longer, respectively, than the values extracted for the sample containing PF_6_^−^. This could be due to magnetic nuclei of PF_6_^−^ being located closer to Mn^2+^ and/or due to more residual motion of the smaller, more symmetric PF_6_^−^ anion as compared to TFSI^−^.

#### Ligand identification

**Fig. 2:**
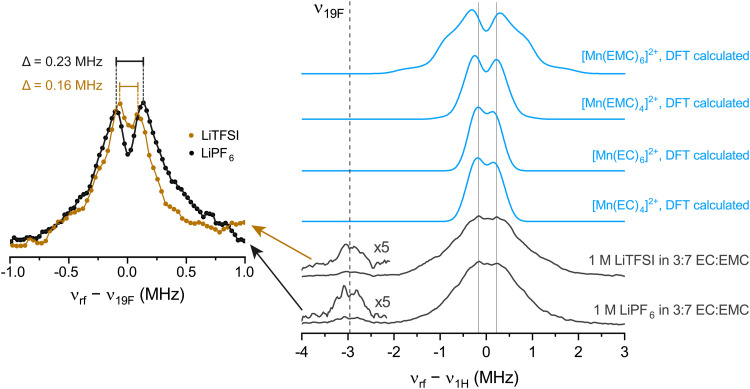
Experimental (black) and simulated (using values extracted from DFT calculations, blue) Davies ENDOR spectra at Q-band microwave frequencies. Excitation *ν*_rf_ was performed at the low-field maximum of the field-swept spectrum shown in Fig. 1b. The radiofrequency (rf) axis is shifted to place the ^1^H or ^19^F Larmor frequency at 0 MHz. The dashed line indicates the ^19^F Larmor frequency at −2.96 MHz (relative to that of ^1^H). Lines at −0.17 and 0.22 MHz are a guide to the eye and centred at the experimental ENDOR maxima. On the left, additional ^19^F Davies ENDOR measurements are shown where the rf axis is shifted to the ^19^F frequency. Interpolation with a cubic spline was used to extract the two local maxima and their frequency difference *Δ*.

To study hyperfine interactions with magnetic nuclei in the first- and second-shell solvation spheres, Davies-type electron nuclear double resonance (ENDOR) spectroscopy at Q-band was applied. Hyperfine couplings of ligand nuclei are typically small compared to their respective nuclear Larmor frequency *ν*_*I*_, and the resonances appear centred around the *ν*_*I*_ specific to each nucleus, exhibiting a powder-like spectral pattern affected by the anisotropy and asymmetry of the hyperfine tensor. The ENDOR experiments reveal couplings to ^1^H and ^19^F nuclei for both samples (Fig. 2). No couplings to ^6,7^Li, ^14^N, or ^31^P were detected, neither in ENDOR nor in additional experiments using other hyperfine-targeted techniques (Fig. S4, ESI†). This implies that these nuclei are likely not within roughly 0.5 nm of the central manganese nucleus. The experimental ENDOR spectra reveal that the largest contribution stems from coupled ^1^H exhibiting a hyperfine coupling of 0.39 MHz, extracted from the local maxima frequency difference, with major shoulders extending up to around ±1 MHz and minor shoulders up to around ±2 MHz. Resonances centred around ν_19F_ are weaker suggesting a significantly smaller quantity of surrounding ^19^F than ^1^H nuclei, even after taking into account signal attenuation of small hyperfine couplings due to a finite microwave pulse length.^54,55^

Given that the ENDOR experiments revealed hyperfine coupling to ^1^H, DFT calculations of Mn^2+^ coordinated to either EC or EMC in fourfold or sixfold complexes were performed, similar to the recent work of some of the authors.^45,46^ Coupling to ^13^C and ^17^O were ignored due to their low natural abundance. Spectra were simulated (Fig. 2, blue traces; Table S2, ESI†) using couplings extracted from the calculations, the simulations representing cumulative contributions from all involved ^1^H nuclei (4 per EC, 8 per EMC), where some conformational variability is intrinsically incorporated through multiple ligands and nuclei. Overall, all the simulated spectra cover a range that is of the same order of magnitude as the experimental spectra. The experimental maxima are best reproduced by EC ligands, but we cannot distinguish between fourfold or sixfold coordination. The shoulders may originate from coordinating EMC, where the flexible side chains can move closer to the central manganese, increasing the hyperfine coupling. However, the shoulders can also be caused by asymmetric strain which is not included in the simulation, other preferred conformers due to effects from outer solvation spheres, or minor impurities like H_2_O ligands which would give rise to intense resonances at around ±1.5 MHz.

Values for the hyperfine couplings of a directly coordinating PF_6_^−^ or F^−^ ion of larger than 10 MHz were estimated from additional DFT calculations (simulations in Fig. S5; parameters in Table S3, ESI†). Thus, the experimental spectra are not consistent with a contact ion pair and the ^19^F nuclei are rather located in anions in the outer solvation sphere. This means that the Fermi-contact interaction can be neglected for ^19^F, leaving a purely dipolar hyperfine tensor. Additional scans in the *ν*_19F_ region reveal weak but distinct local maxima (Fig. 2, left panel) from which an approximate ^55^Mn–^19^F distance can be estimated using *Δ* = *T*, where *T* describes the diagonal hyperfine tensor *A*_19F,diag_ = (−*T*, −*T*, 2*T*). A distance of 6.9 Å for the sample with PF_6_^−^ anions and 7.8 Å for the sample with TFSI^−^ anions is extracted from this tensor. By comparison with the DFT geometry optimised carbonate complex, this distance can be assigned to an anion in the second solvation shell – *i.e.*, the cation–anion distance in an outer sphere complex (Fig. S6, ESI†). The presence of two or more anions in the second coordination shell may be responsible for the *D*/*E* ratio of >0 estimated above from the simulations of the EPR spectra (in Fig. 1). While there may be further, more distant ^19^F ions, resonances with smaller splittings will be even further attenuated, and this estimated distance is likely a good approximation for the closest ^19^F nuclei.

### NMR spectroscopy, 0–60 °C

**Fig. 3:**
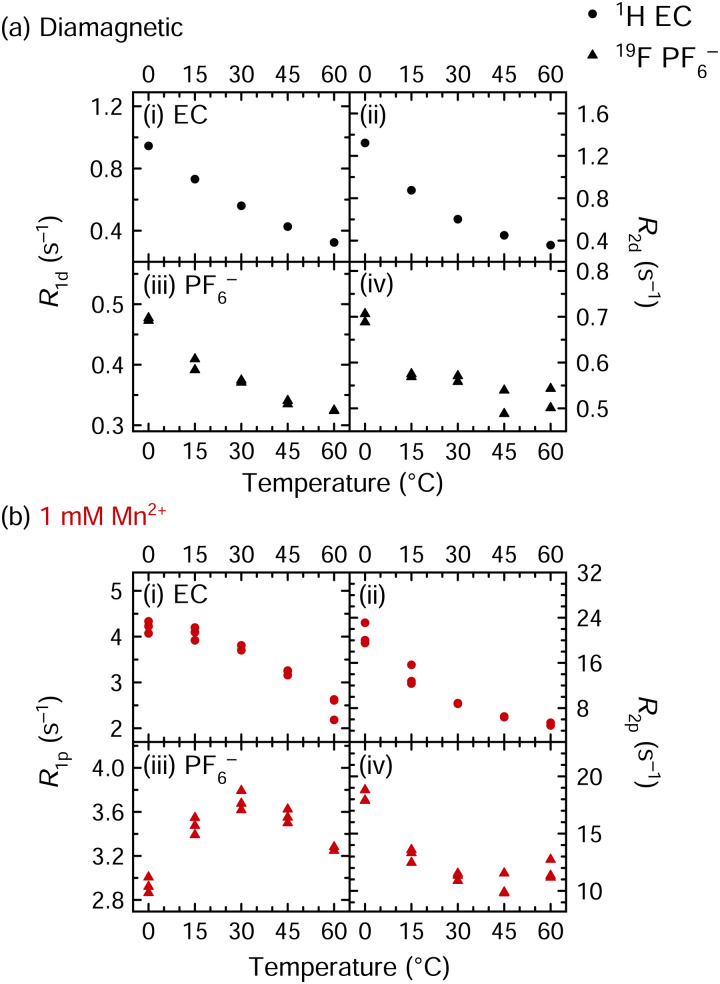
^1^H and ^19^F NMR relaxation rates of (a) diamagnetic and (b) paramagnetic solutions of 1 M LiPF_6_ in 3 : 7 EC : EMC; measurements were performed at a field strength of 11.7 T. Paramagnetic solutions contained 1 mM Mn(TFSI)_2_. Diamagnetic ^1^H EC relaxation, shown in panels (a.i) and (a.ii), contains data from one run, while all other panels contain data from two or three runs.

The EPR measurements provide compelling evidence for no inner-sphere coordination of anions in frozen Mn^2+^ complexes. We now use variable temperature (VT) NMR spectroscopy at 11.7 T to probe solution dynamics in the liquid state. Fig. 3 shows VT relaxation measurements for electrolyte solutions that are either diamagnetic or that contain 1 mM Mn^2+^. The longitudinal and transverse relaxation rates of EC (^1^H) and PF_6_^−^ (^19^F) are shown at 0, 15, 30, 45, and 60 °C. The *R*_1_ and *R*_2_ values of diamagnetic solutions are labelled *R*_1d_ and *R*_2d_, respectively; for paramagnetic solutions, the average *R*_1d_ and *R*_2d_ values are subtracted from measured *R*_1_ and *R*_2_ values to yield *R*_1p_ and *R*_2p_ values, *i.e.*, isolating the relaxation enhancement.

In the diamagnetic solutions, all ^1^H EC and ^19^F PF_6_^−^*R*_1d_ and *R*_2d_ values decrease (become slower) with increasing temperature (Fig. 3a). This decrease with increasing temperature and thus decreasing viscosity, indicates that both ^1^H EC and ^19^F PF_6_^−^ relaxation occur in the fast motion regime. This is consistent with the relatively low viscosity of the electrolyte solutions that are optimised for Li^+^ mobility. Indeed, variable temperature relaxation measurements of a similar electrolyte solution, LiBF_4_ in propylene carbonate, have similarly shown that BF_4_^−^ and propylene carbonate are in the fast motion regime.^86^ For the Mn^2+^-containing solutions, the ^1^H *R*_1p_ and *R*_2p_ values similarly decrease with temperature, indicating that the dynamics that drive relaxation are in the fast regime. The ^19^F *R*_1p_ values initially increase between 0–30 °C then decrease between 30–60 °C. Therefore, at ambient temperature, the relevant mobility process for ^19^F relaxation appears to be in an intermediate regime, with *τ*_c_ of the same order of magnitude as the inverse of the ^19^F Larmor frequency (*i.e.*, 3.4 × 10^−10^ s)^87^ at 11.7 T, whereas the correlation times driving ^1^H relaxation are shorter and in the fast regime. The PF_6_^−^*τ*_c_ value at the *R*_1p_ maximum is consistent with values of *τ*_r_ and *τ*_d_ of ∼10^−9^–10^−10^ s estimated for low viscosity electrolytes.^68^*T*_1e_ values of 6–9 × 10^−6^ s were measured for the Mn^2+^ spins at 20 K; while the *T*_1e_ values are likely shorter at ambient temperature, *τ*_e_ should be the same for EC and PF_6_^−^, *i.e.*, it should not account for the differences in the observed correlation times for ^1^H and ^19^F, suggesting that it is caused by different rotational processes or *τ*_M_ values arising from different binding and exchange processes.

The ^19^F *R*_2p_ values reach a minimum at 45 °C and then increase again. This suggests that the ^19^F PF_6_^−^ paramagnetic relaxation may be exchange-limited in the probed temperature range. Notably, as the different correlation times typically show a different temperature dependence, the dominant contribution may change as the temperature is altered, with *R*_1p_ and *R*_2p_ affected differently. Additionally, if exchange is in the intermediate regime, then *R*_2M_ can be strongly enhanced. According to the SBM model and as discussed further below, these results imply that the *R*_2M_ values likely contain both dipolar and contact terms, while *R*_1M_ is dominated by the dipolar term. A more detailed analysis of the variable temperature ^19^F data is presented in the ESI,† since it does not unambiguously identify the driving forces for relaxation and rather motivates further relaxation experiments described in the next section.

### NMR spectroscopy, ambient temperature

In this section, we separately analyse the trends in the relaxation rate of each environment as the solution composition is changed. We note that ambient temperature measurements were performed at 7.05 T, while VT NMR was performed at 11.7 T. The coordination of both Mn^2+^ and Ni^2+^ is investigated; unlike with pulsed EPR, the rapid Ni^2+^ electronic relaxation and its integer spin does not prevent NMR measurement. Coordination to EMC is not explored directly. We note that metal–solvent coordination is thought to primarily involve EC, because: (i) the solvent properties of EC suggest it is more coordinating than EMC, based on their respective dielectric constants^88^ and solvent polarity parameters (*E*_T_(30) and *E*^*N*^_T_);^89^ and (ii) Li^+^ coordination studies clearly show that EC is preferred over EMC.^7,9,13,15,17^ Additionally, a computational study of Mn^2+^ coordination has shown that EC is preferred over EMC.^21^ That is not to say that no coordination to EMC can occur: our previous NMR studies of 1 M LiPF_6_ in 3 : 7 EC : EMC containing dissolved Mn(TFSI)_2_ showed a larger ^1^H hyperfine shift for the EC resonance than for all EMC resonances, suggesting that while coordination to EC is likely preferred, coordination to EMC is also possible.^22^ Some variation likely exists among the coordination environments of paramagnetic ions, with several possible solvation environments of varying probabilities—but the fraction of Mn^2+^ or Ni^2+^ coordinated to EMC is probably small (or the fraction of time that an EMC molecule spends coordinated to a transition metal is small). Ambient temperature EMC relaxation data are, however, provided in the ESI.†

#### Viscosity measurements

To explore the coordination between transition metal ions and the different electrolyte components *via* NMR, solutions were prepared with constant transition metal concentrations but with varying concentrations of LiPF_6_, EC, and LiTFSI. Notably, changing the concentration of these components affects the overall solution viscosity. The nuclear relaxation behaviour of both diamagnetic and paramagnetic solutions depends in part upon the rotational and diffusional correlation times, and both the rotation of the different complexes and the diffusion of the various species in solution varies with viscosity (eqn (5)). Kinematic viscosities were therefore measured for diamagnetic electrolyte solutions studied in this work (any viscosity change due to the addition of 1 mM Ni(TFSI)_2_ or 1 mM Mn(TFSI)_2_ is assumed to be negligible). Densities were also measured or extracted from the literature and used to convert kinematic viscosities to dynamic viscosities. These values are presented in Table 2.

**Table 2:** Ambient temperature kinematic viscosities, densities, and dynamic viscosities of the electrolyte solutions used in this work. Error in the kinematic viscosities reflects the standard deviation of three measurements. EMC density (marked *) is a reference value;^90^ all other solution densities were measured. Dynamic viscosities were determined by multiplying the kinematic viscosities by the solution densities

	Kinematic viscosity (mm^2^ s^−1^)	Density (g mL^−1^)	Dynamic viscosity (mPa s)
EMC	0.617 ± 0.003	1.012*	0.62
3 : 7 EC : EMC (v/v)	0.940 ± 0.002	1.11	1.05
1 M LiPF_6_ in EMC	1.440 ± 0.006	1.10	1.58
1 M LiTFSI in 3 : 7 EC : EMC	2.183 ± 0.005	1.23	2.68
1 M LiPF_6_ in 3 : 7 EC : EMC	2.518 ± 0.007	1.20	3.03

Both the kinematic and dynamic viscosities follow the order: 1 M LiPF_6_ in 3 : 7 EC : EMC (v/v) > 1 M LiTFSI in 3 : 7 EC : EMC > 1 M LiPF_6_ in EMC > 3 : 7 EC : EMC > EMC. The values presented here are consistent with literature values for the dynamic viscosity of EMC (0.65 cP at 25 °C), 3 : 7 EC : EMC w/w (1.11 cP at 20 °C), and 1 molal LiPF_6_ in 3 : 7 EC : EMC w/w (3.05 cP at 20 °C).^91^

#### Nuclear relaxation measurements: coordination to EC and PF_6_^−^

**Fig. 4:**
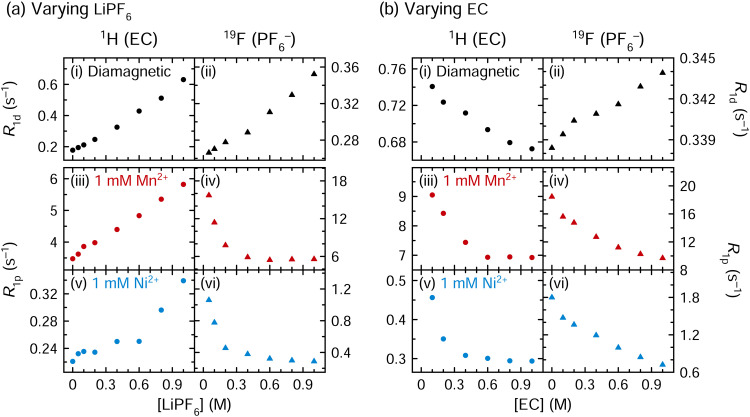
The effect of (a) LiPF_6_ and (b) EC concentration on longitudinal nuclear relaxation rates. Panels show (i), (iii) and (v) ^1^H EC (circles) and (ii), (iv) and (vi) ^19^F PF_6_^−^ (triangles) relaxation rates of diamagnetic, Mn^2+^-containing, and Ni^2+^-containing solutions of 3 : 7 EC : EMC (v/v) with 0–1 M LiPF_6_ or 1 M LiPF_6_ in EMC with 0–1 M EC. *R*_1d_ (i) and (ii) indicates the *R*_1_ value of diamagnetic solutions, while *R*_1p_ (iii)–(vi) indicates the paramagnetic relaxation enhancement (*R*_1p_ = *R*_1_ − *R*_1d_). (Note: 3 : 7 EC : EMC (v/v) contains 4.5 M EC.) All measurements were performed at a field strength of 7.05 T.

Fig. 4 shows the longitudinal relaxation rates of electrolyte solutions as the LiPF_6_ or the EC concentration is increased from 0 to 1 M (*i.e.*, 3 : 7 EC : EMC + 0–1 M LiPF_6_ and 1 M LiPF_6_ in EMC + 0–1 M EC). Solutions contain either no transition metals (diamagnetic) or 1 mM Mn^2+^ or Ni^2+^ (paramagnetic).

For solutions with varying LiPF_6_, in the diamagnetic solutions, ^1^H EC and ^19^F PF_6_^−^ relaxation rates both increase as the PF_6_^−^ concentration increases (Fig. 4a-i and ii). In the paramagnetic solutions, the ^1^H EC *R*_1p_ values increase as the PF_6_^−^ concentration increases, but the ^19^F PF_6_^−^*R*_1p_ values decrease. As LiPF_6_ is added to 3 : 7 EC : EMC, the solution becomes significantly more viscous (Table 2), increasing the rotational and diffusional correlation times of the species in solution. In the fast motion regime, longer correlation times increase the relaxation rate, until an *R*_1_ maximum (or *T*_1_ minimum) occurs.^68,92^ In the paramagnetic solutions, the ^1^H EC relaxation rates increase as the PF_6_^−^ concentration increases (Fig. 4a-iii and v); this change is the same as the diamagnetic solution and again is likely a viscosity effect.

The paramagnetic contribution to the longitudinal relaxation rate, *R*_1p_, is proportional to the fraction of nuclei bound to paramagnetic ions, and it is also dependent on the length of time the nuclei are nearby paramagnetic ions (eqn (9)). The decrease in ^19^F PF_6_^−^ relaxation rates as the PF_6_^−^ concentration increases (Fig. 4a-iv and vi) is consistent with the reduction in the ratio of transition metal ions to PF_6_^−^. Assuming the total number of paramagnetic-coordinated PF_6_^−^ ions remains constant, as the overall molar fraction of PF_6_^−^ grows, the coordinated fraction of PF_6_^−^ becomes smaller. For instance, if Mn^2+^ is ordinarily nearby two PF_6_^−^ ions, then as the PF_6_^−^ concentration changes from 0.05 M to 1 M, in a solution containing 1 mM Mn^2+^, the Mn^2+^-coordinated fraction of PF_6_^−^ would decrease from 4% to 0.2%. The PF_6_^−^ molecule is less likely, on average, to be bound or in close proximity to a paramagnetic ion, *e.g.*, in a second coordination shell; hence, the overall PF_6_^−^ relaxation rate is decreased. The observed trend is not linear, which is ascribed, at least in part, to the simultaneous viscosity increase as LiPF_6_ is added to solution. EMC relaxation rates are not discussed in detail here; however, the ^1^H EMC relaxation rates do increase in diamagnetic and paramagnetic solutions as LiPF_6_ is added, presumably due to the viscosity change (Fig. S8, ESI†).

The ^1^H EC and ^19^F PF_6_^−^ relaxation rates of diamagnetic and paramagnetic solutions were also measured as the EC concentration in solution was increased from 0–1 M, with the LiPF_6_ concentration constant at 1 M (Fig. 4b). Increasing the EC concentration increases the solution viscosity (Table 2): thus, in the diamagnetic case, the ^19^F PF_6_^−^ relaxation rates increase (as do the ^1^H EMC relaxation rates, shown in Fig. S9, ESI†). However, the diamagnetic ^1^H EC relaxation rate decreases as the EC concentration increases. The EC relaxation is therefore controlled by a different (non-viscosity) mechanism, which is likely related to the extent of Li^+^ coordination: while 3 : 7 EC : EMC (v/v) contains 4.5 M EC, the solutions in Fig. 4b contain only 0–1 M EC. Li^+^ is preferentially solvated by EC over EMC,^7,9,13,15,17^ but in these solutions, 1 M Li^+^ cannot be fully (tetrahedrally) solvated by EC and there is likely no free EC. Rather, many Li^+^ ions are left to compete for solvation by EC, and with ≤1 EC available per Li^+^, the binding interaction may be stronger than in a solution with a larger EC concentration, as each Li^+^ does not have any other EC molecules to bind to, and the rate of EC exchange in the Li^+^ solvation shell is likely slower. Thus, an EC molecule in a solution of 0–1 M EC + 1 M LiPF_6_ on average is more likely to exist as a bound Li^+^–EC complex, relative to an EC molecule in a solution of 4.5 M EC + 1 M LiPF_6_ where free EC molecules are also present. Since the Li^+^–EC complex is larger than a free EC molecule, it is associated with longer rotational correlation times than free EC, even though the overall solution is less viscous.

In the paramagnetic solutions, the ^1^H EC and ^19^F PF_6_^−^ relaxation rates both decrease as the EC concentration increases (Fig. 4b-iii–vi), neither being consistent with a (dominant) viscosity-driven inner sphere or an outer sphere mechanism. The decrease in ^1^H EC relaxation rate as EC is added to the solution is consistent with both the diamagnetic case, as well as eqn (9), which predicts slower ^1^H EC relaxation as the metal : EC ratio changes from 1 : 100 to 1 : 1000 (due to the smaller *f*_M_, the EC spending less time on average bound to a paramagnetic ion). The ^19^F PF_6_^−^ relaxation time decreases as EC is added, likely because EC is preferentially adopted into the transition metal coordination sphere, and *f*_M_ is thereby reduced. When EC is absent, PF_6_^−^ spends more time in the transition metal inner coordination shell, resulting in the fastest PF_6_^−^ relaxation rates in EC-free solution. By contrast, when the EC concentration is increased further from 1 M to 4.5 M, or 3 : 7 EC : EMC (shown in Fig. 4a, highest concentration point), the ^19^F *R*_1p_ values drop from 9.7 to 5.6 s^−1^ for Mn^2+^ and from 0.72 to 0.29 s^−1^ for Ni^2+^, consistent with the predominance of EC in the Mn^2+^ inner shell seen by EPR.

Taken together, the results in Fig. 4a and b support the EPR results, showing that Mn^2+^ and Ni^2+^ coordinate preferentially to EC over PF_6_^−^. Removal of PF_6_^−^ from solution affects the EC relaxation rate only little, and it is still dominated by a viscosity effect: if PF_6_^−^ coordination were preferred, removing PF_6_^−^ would increase the M^2+^–EC fraction, which may produce a faster ^1^H EC relaxation rate in LiPF_6_-free solution. Instead, the opposite is observed (Fig. 4a-i, iii and v), due to the higher viscosity of LiPF_6_-containing solution. By contrast, removing EC from solution causes a dramatic increase in the PF_6_^−^ relaxation rate (Fig. 4b-ii, iv and vi), which is notably opposite to what the viscosity change would predict: this indicates that M^2+^–PF_6_^−^ coordination is favoured only in the absence of EC, and that the addition of EC reduces the M^2+^–PF_6_^−^ fraction.

**Fig. 5:**
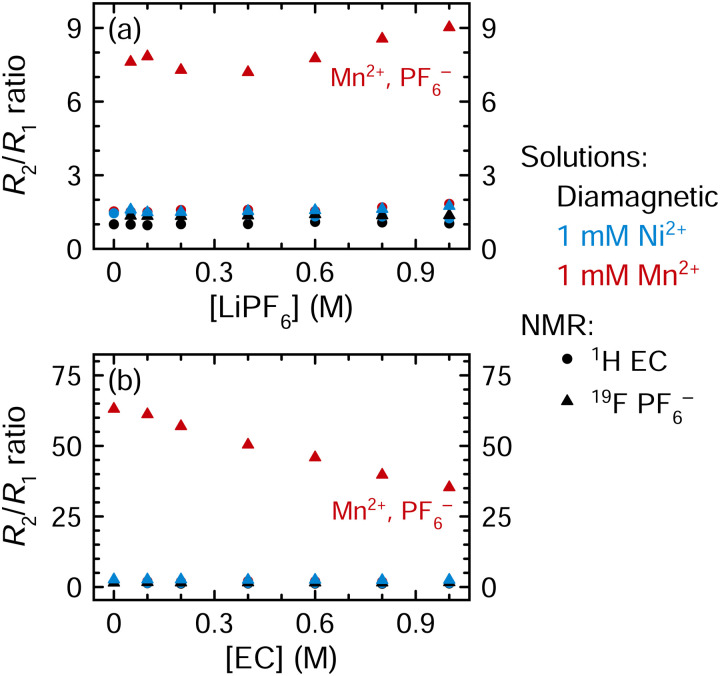
*R*_2_/*R*_1_ ratios of (a) solutions of 3 : 7 EC : EMC (4.5 M EC) with 0–1 M LiPF_6_ or (b) solutions of 1 M LiPF_6_ in EMC with 0–1 M EC. Solutions are diamagnetic or contain Ni^2+^ or Mn^2+^; relaxation of ^1^H EC (circles) and ^19^F PF_6_^−^ (triangles) is shown. Expanded view of small *R*_2_/*R*_1_ ratios is shown in ESI,† Fig. S10. Measurements were performed at a field strength of 7.05 T.

Fig. 5 shows the *R*_2_/*R*_1_ ratios for all solutions examined in Fig. 4 (an expanded view showing small *R*_2_/*R*_1_ ratios is shown in Fig. S10, ESI†). The *R*_2_/*R*_1_ ratios are small for the ^1^H EC resonance for all Mn^2+^-containing samples (1.5–2.1) and for all resonances of the Ni^2+^-containing solutions. In contrast, the *R*_2_/*R*_1_ ratios are consistently large for the ^19^F PF_6_^−^ resonance in all Mn^2+^-containing samples. When the LiPF_6_ concentration is varied (Fig. 5a), the ^19^F PF_6_^−^*R*_2_/*R*_1_ ratio is ∼8, but when the EC concentration is varied (Fig. 5b), the ^19^F PF_6_^−^*R*_2_/*R*_1_ ratio decreases linearly from 63.1 to 35.3 as the EC concentration increases to 1 M; it drops further to 9.0 when EC is present at 4.5 M (Fig. 5a, highest concentration point).

The ambient temperature NMR measurements were performed at a lower field strength of 7.05 T, compared to 11.7 T for VT NMR measurements. As a result, ^1^H relaxation rates are now more distinctly within the fast regime, while the ^19^F *R*_1_ rates are also shifted towards the fast regime, since *R*_1_ maxima are now expected at lower temperatures at the lower field. Assuming that the ^19^F relaxation is close to a maximum for 1 M LiPF_6_ in 3 : 7 EC : EMC—and in the fast regime—then if the EC concentration is decreased, which should reduce viscosity and *τ*_r_*, R*_1p_ is predicted to decrease. Instead *R*_1p_ is seen experimentally to increase (Fig. 4), which we ascribed earlier to the decrease of EC bound to the paramagnetic ions in the inner coordination shell. Furthermore, the LiPF_6_^19^F *R*_2_/*R*_1_ ratio increases noticeably from 9 in 3 : 7 EC : EMC electrolytes to 63 in EMC-only electrolytes (Fig. 5). In an outer sphere mechanism, *R*_1_ and *R*_2_ should approach each other in the fast regime. This suggests that a driving force (fluctuating field) beyond simply an outer-shell dipolar mechanism plays an increasingly important role, at least at low EC concentrations. We suggest that inner sphere mechanisms start to play a larger role; this is unsurprising because less EC is present in the Mn^2+^ inner shell.

In an inner sphere complex, Mn^2+^ will coordinate directly to ^19^F, resulting in more electron density at the nucleus being studied, and a large hyperfine interaction (static average DFT value of *A*_iso_(^19^F) = 2.90 MHz including Mn–F–P, Table S3, ESI†). Thus, at lower EC concentrations, at least, we tentatively suggest the large *R*_2_/*R*_1_ ratios may arise from a contact contribution to relaxation for ^19^F. Rotations of the coordinating PF_6_^−^ anion, leading to changes in the F atoms that are coordinated to Mn^2+^, will also result in large fluctuations of the ^19^F hyperfine interactions, providing another (contact) relaxation mechanism. By contrast, with EC, Mn^2+^ coordinates at the carbonyl oxygen,^20,21^ which is located much farther away from the ^1^H nuclei that are being studied, resulting in a small hyperfine interaction as is seen in the EPR results (static average DFT value of *A*_iso_(^1^H) = 0.03 MHz, Table S2, ESI†) and thus a small contact term for ^1^H. The observed small ^1^H *R*_2_/*R*_1_ ratios are consistent with this.

#### Nuclear relaxation measurements: coordination to TFSI^−^

**Fig. 6:**
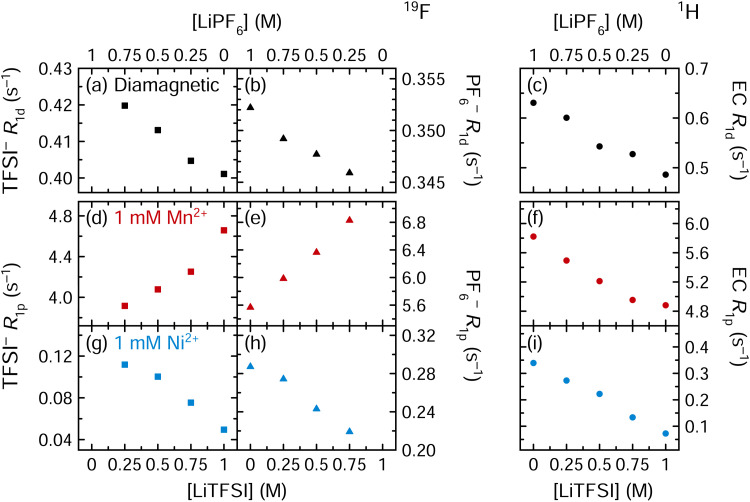
(a), (d) and (g) ^19^F TFSI^−^ (squares), (b), (e) and (h) ^19^F PF_6_^−^ (triangles), and (c), (f) and (i) ^1^H EC (circles) longitudinal relaxation rates of solutions of 3 : 7 EC : EMC (v/v) with 0–1 M LiPF_6_ and 1–0 M LiTFSI, where the total Li^+^ concentration remained constant at 1 M. Solutions were diamagnetic or contained 1 mM Mn(TFSI)_2_ or Ni(TFSI)_2_.

Measurements to assess the degree of coordination between the transition metal cations and the TFSI^−^ counterion were then performed. Fig. 6 shows the effect of incrementally replacing LiPF_6_ with LiTFSI; the salt concentration in solution is 1 M in total, comprising either 0%, 25%, 50%, 75%, or 100% LiTFSI, with the remainder of the salt content comprising LiPF_6_.

In Fig. 6a–c, the diamagnetic relaxation rates all decrease as the LiPF_6_ salt is replaced by LiTFSI; this is consistent with the viscosity measurements of these solutions in Table 2 showing that the LiPF_6_ solution is more viscous. When the solution contains 1 mM Mn^2+^, the ^1^H EC relaxation rates decrease (Fig. 6f), again matching the diamagnetic, viscosity-driven behaviour. However, the ^19^F PF_6_^−^ and ^19^F TFSI^−^ relaxation rates both increase as LiPF_6_ is replaced by LiTFSI (Fig. 6d and e). If Mn^2+^ coordination to PF_6_^−^ and TFSI^−^ are equally preferable, there should be no effect, to a first approximation, with changing the LiPF_6_/LiTFSI concentration, as the ratio of Mn^2+^-coordinated anions *vs.* total anions would stay the same. However, if PF_6_^−^ coordination is preferred over TFSI^−^, then the ^19^F PF_6_^−^*R*_1p_ and ^19^F TFSI^−^*R*_1p_ values should both increase as LiTFSI replaces LiPF_6_, as is observed. (If PF_6_^−^ coordination is preferred, further increasing the PF_6_^−^ concentration only reduces the total coordinated fraction, lowering PF_6_^−^*R*_1p_ values.) Notably, this result is inconsistent with previous computational work suggesting TFSI^−^ coordination should be preferred.^21^ However, variable temperature NMR measurements were not performed for TFSI^−^ solutions, and it is possible that the paramagnetic ^19^F TFSI^−^*R*_1_ is exchange-limited. If so, then increasing the TFSI^−^ concentration may result in more rapid TFSI^−^ chemical exchange, which could ultimately cause the relaxation rate to increase (*via*eqn (9)). In outer sphere mechanisms, since the concentration of metals remains constant, the viscosity of the solutions is expected to dominate this mechanism, the results lending further support to the role that there is an inner sphere contribution to relaxation.

In the Ni^2+^-containing solutions (Fig. 6g–i), the ^19^F TFSI^−^, ^19^F PF_6_^−^, and ^1^H EC relaxation rates all decrease as LiPF_6_ is replaced by LiTFSI. While these are the same results as observed in diamagnetic solution, this is not explained entirely by viscosity effects: Fig. 4a-vi shows that in a Ni^2+^-containing solution, removing PF_6_^−^ (without replacing it with TFSI^−^) does cause the ^19^F PF_6_^−^*R*_1p_ values to increase as *f*_M_ increases. The largest ^19^F PF_6_^−^*R*_1p_ value coinciding with the smallest Ni^2+^ : PF_6_^−^ ratio (1 : 1000) therefore suggests that Ni^2+^ is only nearby PF_6_^−^ when PF_6_^−^ is present in substantial concentrations. Additionally, the decrease in ^19^F TFSI^−^*R*_1p_ values as the TFSI^−^ concentration increases is consistent with a decrease in *f*_M_. Fig. 6 therefore indicates that Ni^2+^ prefers TFSI^−^ coordination over PF_6_^−^ coordination, while Mn^2+^ prefers PF_6_^−^ coordination over TFSI^−^ coordination. However, in a 1 M LiPF_6_ electrolyte solution where 1 mM Mn(TFSI)_2_ or Ni(TFSI)_2_ is added as a model compound, the PF_6_^−^ concentration is 500× larger than the TFSI^−^ concentration and interactions with the PF_6_^−^ anion may dominate for both Mn^2+^ and Ni^2+^.

An analysis of the *R*_2_/*R*_1_ ratios for the same solutions as studied in Fig. 6 is provided in the ESI† (Fig. S11); these data are consistent with conclusions drawn from the *R*_1p_ data. The idea of preferential coordination to TFSI^−^ by Ni^2+^ but not Mn^2+^ may be rationalised on the basis of crystal field arguments. Ni^2+^ (d^8^) complexes, with larger crystal field stabilisation energies and smaller radii, are more long lived once they form, whereas Mn^2+^ (d^5^) complexes have zero crystal field stabilisation energies, larger radii, and more rapid equilibria (following the Irving–Williams order of stability), *i.e.*, more fluctional, short-lived complexes are formed.^93–95^ The more rapid equilibria found for Mn^2+^ ions means that EC molecules will also move in and out of the Mn^2+^ coordination shell, allowing Mn^2+^–PF_6_^−^ inner sphere interactions to occur, albeit short-lived, contributing to the more rapid transverse nuclear relaxation. These interactions are weak, however, so that no inner sphere binding is seen in the EPR experiments. Thermodynamically, the M^2+^–TFSI^−^ interaction is likely stronger than the M^2+^–PF_6_^−^ interaction, in accordance with computational work for Mn^2+^,^21^ but transition metals may coordinate at any F of the small, symmetric PF_6_^−^ molecule, whereas TFSI^−^ is bulkier and may be more difficult to accommodate around a metal ion. In actual electrolyte solutions containing dissolved transition metals and multiple solvent molecules, it may be that the smaller PF_6_^−^ is easier to incorporate in the Mn^2+^ solvation shell and thus outcompete TFSI^−^ in this respect. By contrast, the greater crystal field stabilisation of Ni^2+^ should result in stronger binding of TFSI^−^ to Ni^2+^*vs.* binding to Mn^2+^, combined with longer lived complexes. These results indicate the importance of experimental studies to complement theoretical work. Finally, we note that the timescales in which the anions and solvation molecules move in and out of the solvation shells, and the role that metal binding has on the rotational modes of the anions themselves, will clearly have implications for the different relaxation processes, motivating further molecular dynamics simulations of these systems, coupled with more variable temperature NMR studies.

## Conclusions

Electron–nuclear spin interactions have been exploited in this study to identify the solvation sphere(s) of paramagnetic transition metal ions in battery electrolytes, combining synergetic insights from EPR and NMR into ligand identity and dynamics. Pulsed EPR spectroscopy was beneficial for the direct observation of ligands using ENDOR. However, EPR is limited to systems with slow electronic relaxation rates, typically requiring cryogenic measurement temperatures, and is best suited to metals with non-integer electron spins, *i.e.*, Mn^2+^. In contrast, simple *T*_1_ and *T*_2_ NMR measurements at ambient conditions can provide indirect insights into the nature of solvation shells, for a wide range of paramagnetic ions and including dynamic effects such as ligand exchange. This is due to nuclear relaxation rates of electrolyte components being highly sensitive to the presence of paramagnetic transition metals.

In frozen pristine electrolyte solutions, EPR experiments identified that dissolved Mn^2+^ is primarily coordinated to EC in solutions of 1 M LiPF_6_ or LiTFSI in 3 : 7 EC/EMC (v/v), with distorted *O*_h_ symmetry (sixfold coordination). In addition to EC coordination, rhombic zero-field splitting suggests an asymmetric coordination environment, influenced by EMC and/or salt anions. This is consistent with NMR experiments of Mn^2+^- and Ni^2+^-containing solutions with a variety of salt and solvent concentrations. NMR results showed that EC outcompetes PF_6_^−^ in the solvation shell. At the same time, the extremely fast ^19^F PF_6_^−^ transverse relaxation in Mn^2+^-containing solutions, particularly at low EC concentrations, likely arises from a contact term and indicates that some PF_6_^−^ is indeed present in the first solvation shell (*i.e.*, as contact ion pairs). In contrast, ^19^F ENDOR experiments do not indicate the presence of a contact ion pair, but instead a solvent-separated ion pair is found, presumably due to the sole presence of the thermodynamically more stable EC-coordinated complex at cryogenic temperature. Taken together, the results suggest that the Mn^2+^ and Ni^2+^ solvation shell in pristine electrolyte solutions comprises primarily EC, with some exchange of PF_6_^−^ between the inner and outer spheres, particularly in Mn^2+^ solutions at low EC concentrations. NMR experiments probing coordination to TFSI^−^ showed that TFSI^−^ can displace PF_6_^−^ in the Ni^2+^ solvation shell, but there is no clear evidence from either NMR or EPR that this occurs in the Mn^2+^ solvation shell.

These new insights on transition metal coordination add experimental evidence to previous work, which is dominated by computational studies. A clear understanding of metal solvation may contribute to approaches adopted to prevent transition metal dissolution, deposition, and overall battery capacity fade. Finally, the presented combined EPR–NMR approach is well-suited to assess the solvation of paramagnetic transition metals and may be readily applied to any other electrolyte system or cell chemistry, including studying transition metal coordination to novel electrolyte components or electrolyte degradation species.

## Data availability

Additional data supporting this article, including DFT xyz files, have been included as part of the ESI.†

## Conflicts of interest

There are no conflicts to declare.

## Supplementary Material

CP-026-D4CP01663G-s001

CP-026-D4CP01663G-s002

CP-026-D4CP01663G-s003
