# Supplementary material for: Coordination of dissolved transition metals in pristine battery electrolyte solutions determined by NMR and EPR spectroscopy
Source: Phys Chem Chem Phys. 2024 Jul 9;26(28):19505–20. doi: 10.1039/d4cp01663g (PMC11253248; doi:10.1039/d4cp01663g)
Supplement: CP-026-D4CP01663G-s003 [file CP-026-D4CP01663G-s003.pdf]

Electronic Supplementary Material (ESI) for Physical Chemistry Chemical Physics.
This journal is © the Owner Societies 2024

Coordination of dissolved transition metals in pristine battery electrolyte solutions determined by NMR and EPR spectroscopy

Jennifer P. Allen, Conrad Szczuka, Holly E. Smith, Erlendur Jónsson, Rüdiger-A. Eichel, Josef Granwehr, Clare P. Grey

Supplementary Information

DFT

Prior to tackling the DFT calculations of the EPR shifts, a series of complexes, $[Mn(EC)_x]^{2+}$ (x=1,...,8), were constructed to see effect of increasing solvation on the energetics of the EC-solvation of Mn^{2+}. Initial structures were the idealised forms, e.g. tetrahedral for x = 4 and trigonal bipyramidal for x = 5. In each case, the structures had their geometry optimised with the B3LYP functional (D3BJ dispersion correction) with the def2-TZVP basis set. Orca version 5.0.3 was used throughout this section along with the default RIJCOSX settings. Furthermore, the tight SCF convergence criteria was used along with the enhanced defgrid3 grid settings. After optimisation, frequency calculations were performed to confirm convergence and get thermochemistry results. In some cases, very tight geometry and SCF convergence criteria were required to ensure proper geometry convergence. The results are shown in Table S1.

Table S1. Results of DFT calculations exploring the level of EC-solvation of Mn^{2+}. The $\Delta E_{complex}$ was calculated by $E_{complex}$ - x*E_{EC} - E_{Mn} with stepwise comparison shown with $\partial\Delta E_{complex}$. The Gibbs free energy, $\Delta G_{complex}$, was calculated similarly.

Complex	$\Delta E_{complex}$ / kJ·mol^{-1}	$\partial\Delta E_{complex}$ / kJ·mol^{-1}	$\Delta G_{complex}$ / kJ·mol^{-1}	$\partial\Delta G_{complex}$ / kJ·mol^{-1}
$[Mn(EC)_1]^{2+}$	-572.6		-546.9	
$[Mn(EC)_2]^{2+}$	-984.1	-411.5	-905.9	-359.0
$[Mn(EC)_3]^{2+}$	-1234.0	-249.9	-1094.5	-188.6
$[Mn(EC)_4]^{2+}$	-1423.8	-189.8	-1231.8	-137.3
$[Mn(EC)_5]^{2+}$	-1542.3	-118.5	-1291.7	-59.9
$[Mn(EC)_6]^{2+}$	-1645.2	-102.9	-1333.9	-42.2
$[Mn(EC)_7]^{2+}$	-1694.5	-49.3	-1321.8	12.1
$[Mn(EC)_8]^{2+}$	-1806.4	-111.9	-1372.4	-50.6

As the number of EC molecules increase the electronic and Gibbs free solvation energy of the systems increase, albeit with diminishing returns. Thus, as seen in Table S1, there is a local free energy minimum at x = 6. This was used then as a starting point for an in-depth analysis of EPR data along with additional DFT calculations (described in the main matter).

The optimised $[Mn(EC)_8]^{2+}$ structure had one EC not participating in the solvation of the Mn^{2+}. Instead, this extra EC was interacting with other EC molecules, *i.e.* solvent-solvent interaction, causing a lowering of energy. As the study is on the solvent-metal interaction and as the $[Mn(EC)_7]^{2+}$ system was already shown to have a positive ΔG, further attempts to force a structure without solvent-solvent interactions were abandoned.

EPR

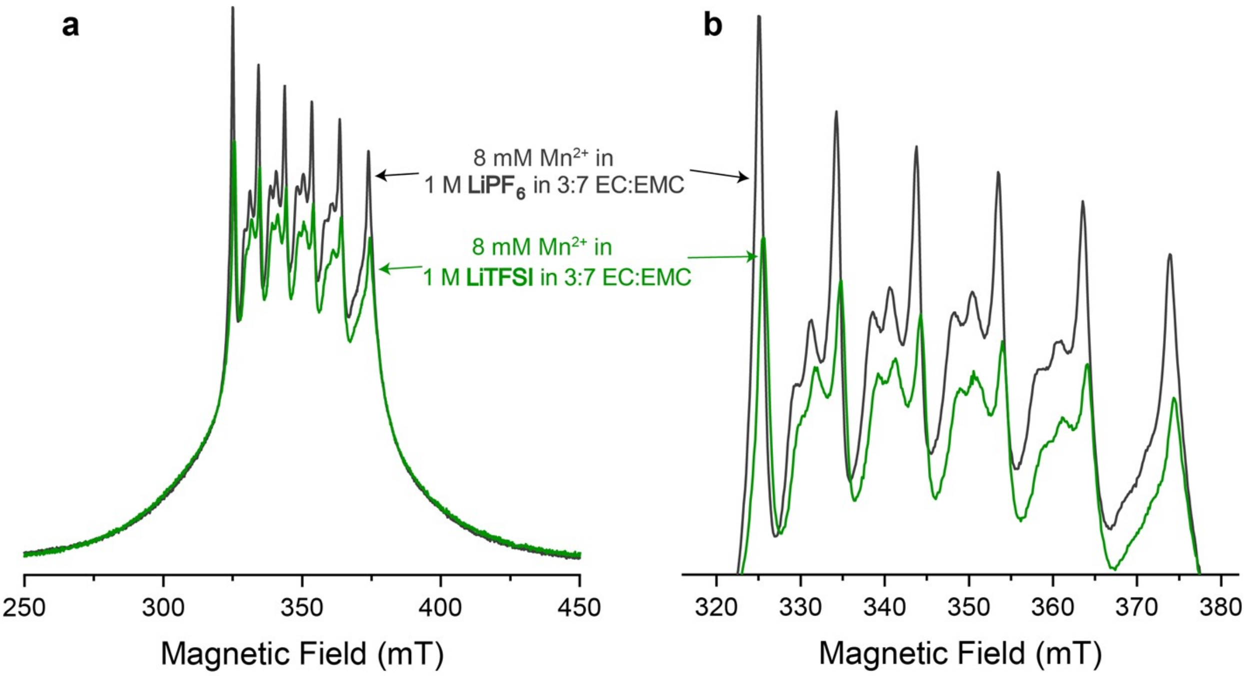

Figure S1. Comparison of experimental pulsed EPR spectra recorded using field-swept Hahn-echoes at X-band. Paramagnetic Mn^{2+} is studied in premixed electrolytes based on $LiPF_6$ or LiTFSI salts. Spectra are normalised to show the resemblance of the outer transitions, characterised by the shoulders of the central transitions. Not exactly overlapping peak positions visible in (**b**) result from slightly different applied X-band microwave frequencies.

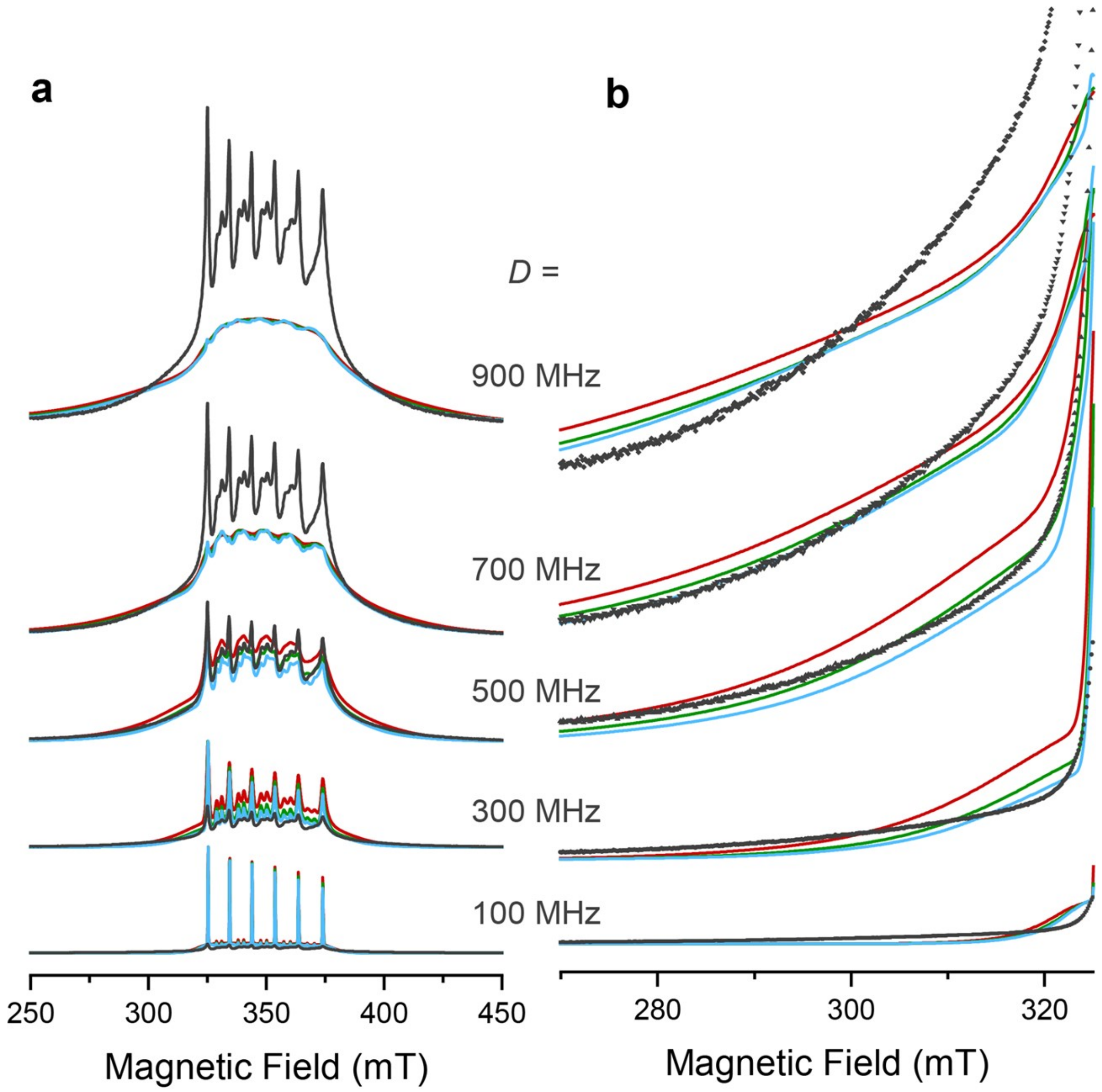

Figure S2. Simulations of EPR spectra with variable zero-field splitting parameters using the *EasySpin* software. Simulations are performed using identical parameters as for Figure 1 in the main text, varying D as indicated and E/D = 0.1 (blue), 0.2 (green), and 0.33 (red). Experimental data of 8 mM Mn^{2+} in 1 M $LiPF_6$ in 3:7 EC:EMC is shown in black for comparison. The D and E strain parameters are assumed to be proportional to the splitting parameters and are set equal to D and E, respectively. These strain values are used to mimic the absence/blurring of additional shoulders at around 300 and 400 mT in the experimental spectrum. The experimental field-swept Hahn-echo spectrum of Mn^{2+} in $LiPF_6$ electrolyte is overlayed for comparison (black) and scaled to match the outer transitions. The full spectra shown in (**a**) reveal that increasing values of D lead to increasingly broadened central transitions and increasing intensity and breadth of the outer transitions. The low-field outer transitions magnified in (**b**) compare the curvature of experiment and simulation. Compared with the least-squares fit referenced in the main text with $|D| \approx 415\ MHz$, the best match lies slightly above 500 MHz, somewhat depending on the chosen E/D. Imperfect fitting of the experimental data can result from a superposition of several similar ligand spheres/conformers of Mn^{2+} complexes or field-dependent T_{2e} dispersion.

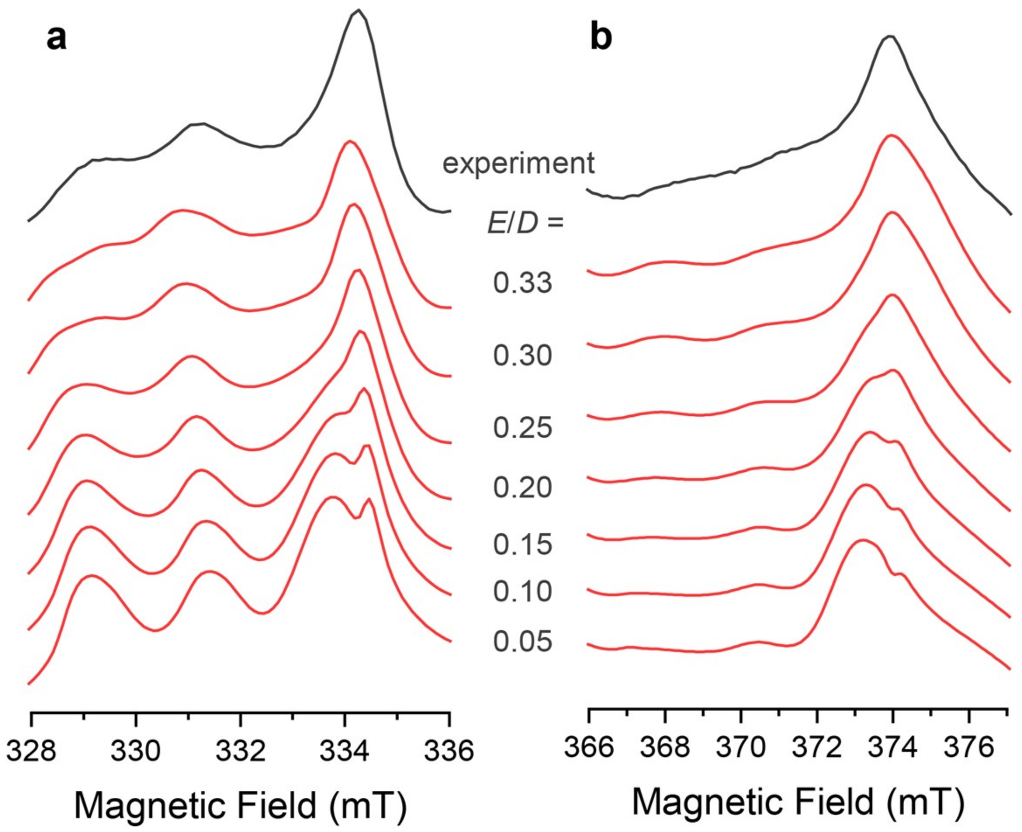

Figure S3. Simulations of EPR spectra with variable zero-field splitting parameter E using the *EasySpin* software (red) in the regions of low- (**a**) and high-field (**b**) central transitions. Experimental data of 8 mM Mn^{2+} in 1 M $LiPF_6$ in 3:7 EC:EMC is shown in black for comparison. Simulations are performed using identical parameters as for Figure 1 in the main text and varying E/D as indicated. The experimental field-swept Hahn-echo spectrum of Mn^{2+} in $LiPF_6$ electrolyte is given in black. Due to accurately determined values for g and $A(^{55}Mn)$, peak positions and intensity ratios are strong indicators for significant rhombicity of the zero-field splitting interaction of the Mn^{2+} complex.

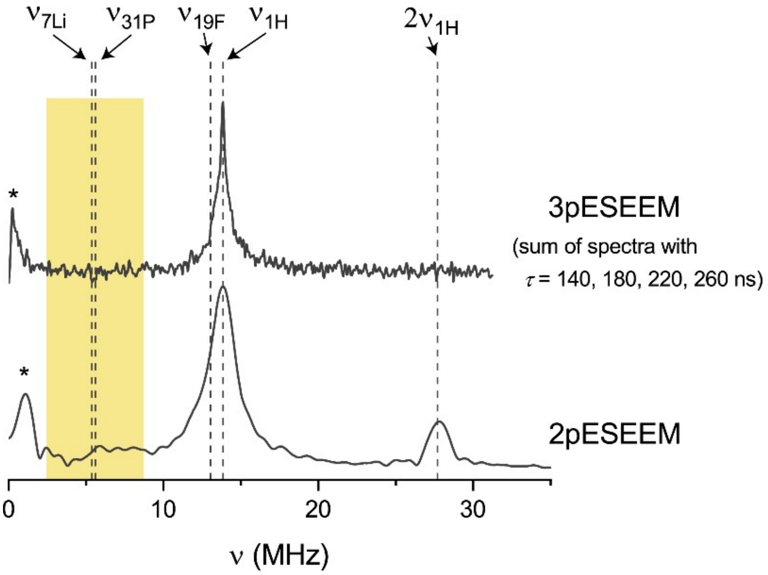

Figure S4. Two-pulse (2p) and three-pulse (3p) electron spin echo envelope modulation (ESEEM) spectra at X-band frequencies of a solution containing 8 mM Mn^{2+} and 1 M $LiPF_6$ in 3:7 EC:EMC. Nuclear Larmor frequencies ν_N with nucleus N at the measurement field of 325 mT are shown as dashed lines. Hyperfine interactions involving ^{7}Li and ^{31}P nuclear spins (in the weak coupling regime) are highlighted in yellow and are absent for this sample. Resonances from ^{14}N are typically also observed in that range, depending on hyperfine and quadrupolar couplings. Asterisks denote artefact peaks from baseline distortions.

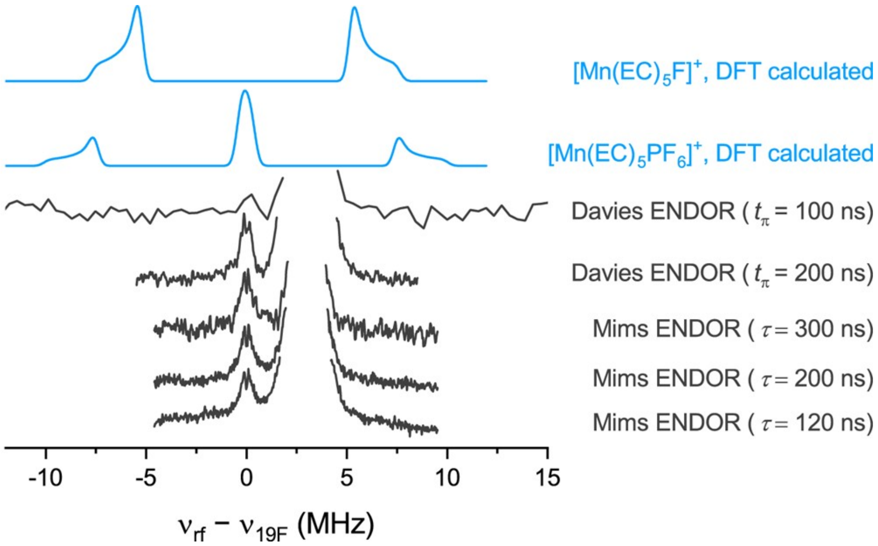

Figure S5. Comparison of experimental (black) and calculated (blue) ENDOR spectra at Q-band frequencies, indicating the absence of fluorine in the first coordination sphere, *i.e.*, directly bound to Mn. Experiments were performed on the sample containing 8 mM Mn^{2+} and 1 M $LiPF_6$ in 3:7 EC:EMC. Several acquisition parameters were used to circumvent influences from spectral blind spots (Mims) and hyperfine contrast selectivity (Davies). The peak around ν_{1H} of the experimental data is truncated for clarity. The traces simulated with coupling constants from DFT calculations in the Davies ENDOR patterns only include the ^{19}F couplings.

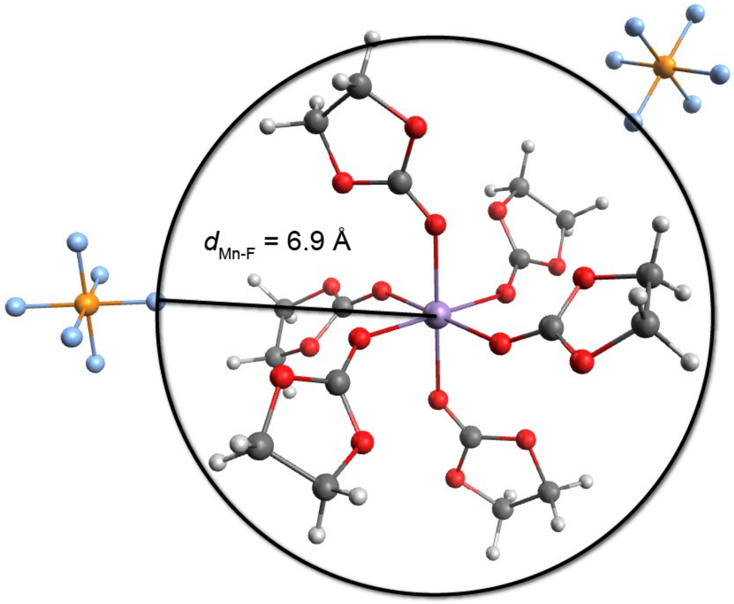

Figure S6. Structure proposition of the ethylene carbonate-separated ion pair of Mn^{2+} with two PF_6^- anions. $[Mn(EC)_6]^{2+}$ atomic positions are taken from geometry-optimised DFT calculations. A separately geometry-optimised PF_6^- anion was added to match the Mn−F distance of 6.9 Å, extracted from the ^{19}F Davies ENDOR measurement. The circle with radius 6.9 Å indicates spherical symmetry of the Davies ENDOR-extracted Mn−F connection vector.

Table S2. DFT-calculated ^{1}H hyperfine coupling constants of $[Mn(EC)_6]^{2+}$. The given order corresponds to the ^{1}H nuclear positions as provided in the xyz-file.

moiety	A_x/MHz	A_y/MHz	A_z/MHz	A_{iso}/MHz
**EC, -CH$_2$-**	-0.302	-0.312	0.672	0.020
	-0.353	-0.365	0.787	0.023
	-0.437	-0.448	0.956	0.024
	-0.408	-0.415	0.901	0.026
	-0.433	-0.443	0.950	0.024
	-0.408	-0.416	0.904	0.027
	-0.303	-0.314	0.672	0.019
	-0.353	-0.366	0.795	0.026
	-0.348	-0.358	0.760	0.018
	-0.293	-0.302	0.694	0.033
	-0.389	-0.396	0.876	0.030
	-0.455	-0.465	0.993	0.024
	-0.389	-0.397	0.877	0.031
	-0.456	-0.466	0.995	0.025
	-0.348	-0.357	0.760	0.018
	-0.293	-0.303	0.695	0.033
	-0.347	-0.357	0.756	0.017
	-0.293	-0.303	0.699	0.035
	-0.390	-0.398	0.880	0.031
	-0.462	-0.473	1.008	0.024
	-0.436	-0.446	0.953	0.024
	-0.405	-0.412	0.895	0.026
	-0.300	-0.311	0.674	0.021
	-0.351	-0.363	0.782	0.022
average				0.025

Table S3. DFT-calculated ^{19}F hyperfine coupling constants of $[Mn(EC)_5PF_6]^+$. The given order corresponds to the ^{19}F nuclear positions as provided in the xyz-file.

moiety	A_x/MHz	A_y/MHz	A_z/MHz	A_{iso}/MHz
**[PF$_6$],** Mn-**F**-P	14.9	15.0	20.2	16.7
**[PF$_6$], F**-P	-0.115	-0.115	0.230	0.000
	-0.122	-0.201	0.778	0.152
	-0.078	-0.141	0.830	0.204
	-0.075	-0.137	0.835	0.208
	-0.124	-0.204	0.774	0.149
average (including Mn-**F**-P)				2.90
average (omitting Mn-**F**-P)				0.142

Variable Temperature NMR

The T_1 minimum, in principle, provides a mechanism for estimating the type and magnitude of the interaction that drives relaxation. To explore this, we modelled the ^{1}H EC and ^{19}F PF_6^- data using Equation 7 from the main text, assuming a single Arrhenius-type behaviour for the correlation time, $\tau_c = \tau_0\, exp\left(\frac{E_a}{RT}\right)$, where E_a is the activation energy of the process driving relaxation, τ_0 is a hypothetical correlation time at infinite temperature, and R is the universal gas constant. Since E_a, τ_0, and the distance r are linearly independent, they can be extracted from a fit of the VT data (Figure S7). For ^{19}F PF_6^- paramagnetic relaxation, values of E_a = 15.2 kJ/mol, τ_0 = 8.5x10^{-13} s, and r = 18.1 Å are obtained. This calculation implicitly assumes that all PF_6^- ions are simultaneously affected by the same dipolar interaction quantified by a single Mn–F distance, r. This assumption was made so as to allow an estimate for the size of the interaction that causes the observed R_{1p} maximum (which is a T_1 minimum). This assumption is clearly not valid, however, given the 1000:1 ratio of PF_6^-:Mn^{2+} ions, but f_M and r cannot be fitted at the same time. An alternative approximation is to use the Mn–F distance at the closest point of approach, r = 6.9 Å, as determined from Davies ENDOR of frozen solutions (Figure S6); this leads to a much shorter T_1 minimum. However, we now must account for the fact that not all PF_6^- can be simultaneously nearby Mn^{2+} and we may attempt to estimate the fraction of coordinated Mn^{2+} coordinated to PF_6^- (*i.e.*, f_M). Using the value of r obtained above (18.1 Å) as an effective distance r_{eff} yields an estimate of f_M of $r^6/r_{eff}^6 \approx 1/325$, which is of the same order of magnitude as [Mn^{2+}]:[PF_6^-]. Or if we assume that two PF_6^- ions are nearby Mn^{2+} for charge balance (in either inner or outer sphere complexes) and f_M = 1/500, then a new value of r of 6.43 Å is obtained. The ^{19}F R_{1p} data are therefore consistent with PF_6^- ions occupying outer sphere sites. Alternatively, we may treat the data with the assumption that an inner sphere complex is present: if on average only 10% of Mn^{2+} ions form an inner sphere complex with PF_6^-, then r = 3.9 Å, which is also not unrealistic. This calculation provides a rough estimate for the concentration of such inner sphere species (which we note were not observed in the ENDOR experiments at 20 K). Since the different relaxation processes may show considerable variations in their activation energies, they will likely depend on sample composition, temperature, or magnetic field. Given the large numbers of approximations and assumptions made to generate these two estimates, either is consistent with EPR and DFT data; an inner sphere complex with low probability could by itself explain the observed ^{19}F R_{1p} results. We suggest that further EPR experiments with different metal-ion and salt concentrations are needed to eliminate the need for some of the approximations.

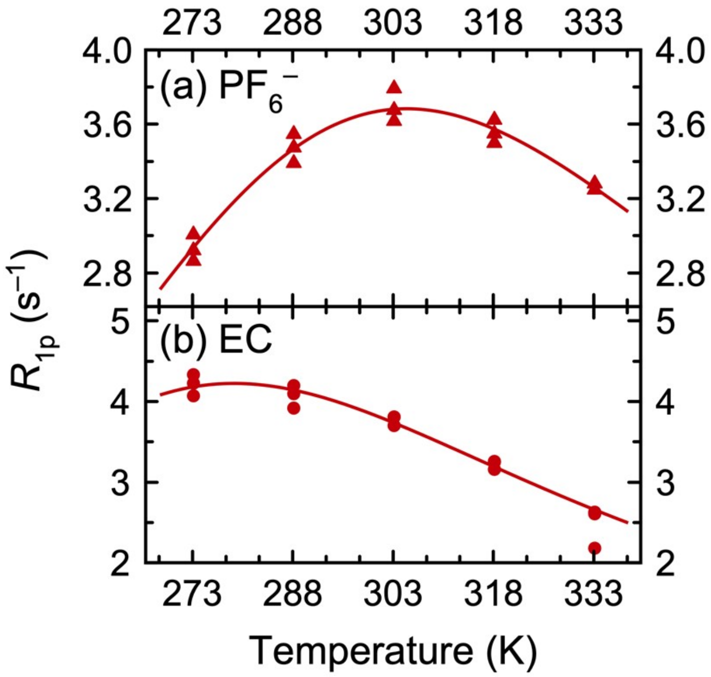

Figure S7. (a) ^{19}F and (b) ^{1}H NMR longitudinal paramagnetic relaxation enhancement of a solution of 1 M $LiPF_6$ in 3:7 EC:EMC + 1 mM $Mn(TFSI)_2$. Diamagnetic data and transverse relaxation rates are also shown in Figure 3. Measurements were performed at a field strength of 11.7 T. Data are fitted using Equation 7 from the main text, assuming a single Arrhenius-type behaviour for the correlation time.

The same approach can be followed to analyse the ^{1}H EC VT R_1 data (Figure S7b). Values of E_a = 14.9 kJ/mol, τ_0 = 5.2x10^{-13} s, and r = 17.9 Å are obtained. In this electrolyte solution, EC is present at 4.5 M. The DFT simulation of $[Mn(EC)_6]^{2+}$ suggests an inner sphere EC coordination with ^{1}H pointing away from the Mn^{2+} ion. Using the average Mn–H distance of r = 5.85 Å, then $f_M \approx 1/820$ is obtained, which is consistent with a theoretical value of $f_M = 1/750$ for Mn^{2+} sixfold coordinated by EC. This estimate for r is likely an upper limit, since in a liquid electrolyte such a complex is flexible, and the distance of closest approach is likely shorter. On the other hand, this flexibility may also hint at a reason for the shorter correlation time found for EC compared to PF_6^-: PF_6^- is a rigid molecule that rotates or moves as a whole, implying a somewhat longer correlation time. However, a very similar activation energy for the relaxation-determining step is found for both ligands, which indicates that a similar overall process may be responsible.

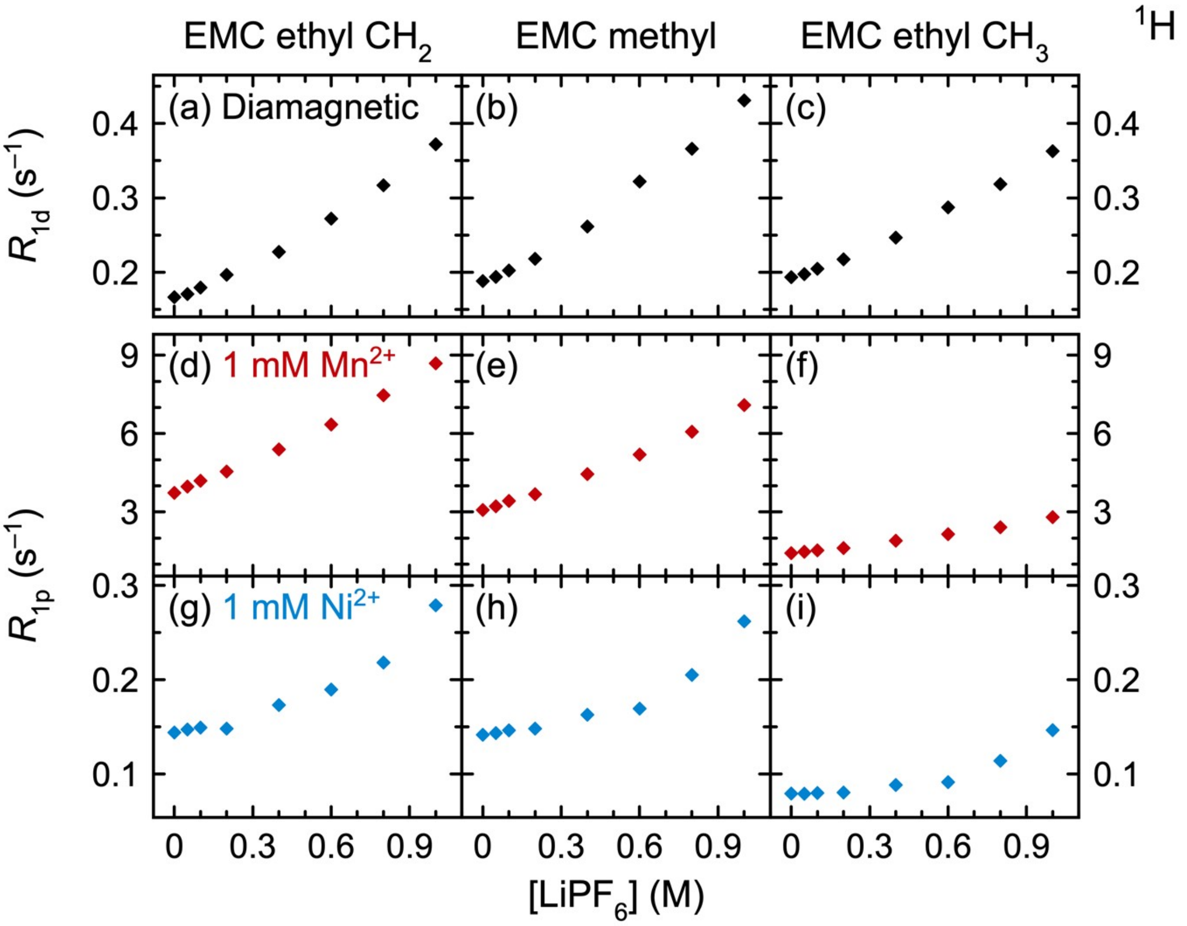

Figure S8. ^{1}H longitudinal nuclear relaxation rates of EMC in (a–c) diamagnetic, (d–f) Mn^{2+}-containing, and (g–i) Ni^{2+}-containing solutions of 3:7 EC:EMC (v/v) with 0–1 M $LiPF_6$. R_{1d} (a–c) indicates the R_1 value of diamagnetic solutions, while R_{1p} (d–i) indicates the paramagnetic relaxation enhancement ($R_{1p} = R_1 - R_{1d}$). All measurements were performed at a field strength of 7.05 T.

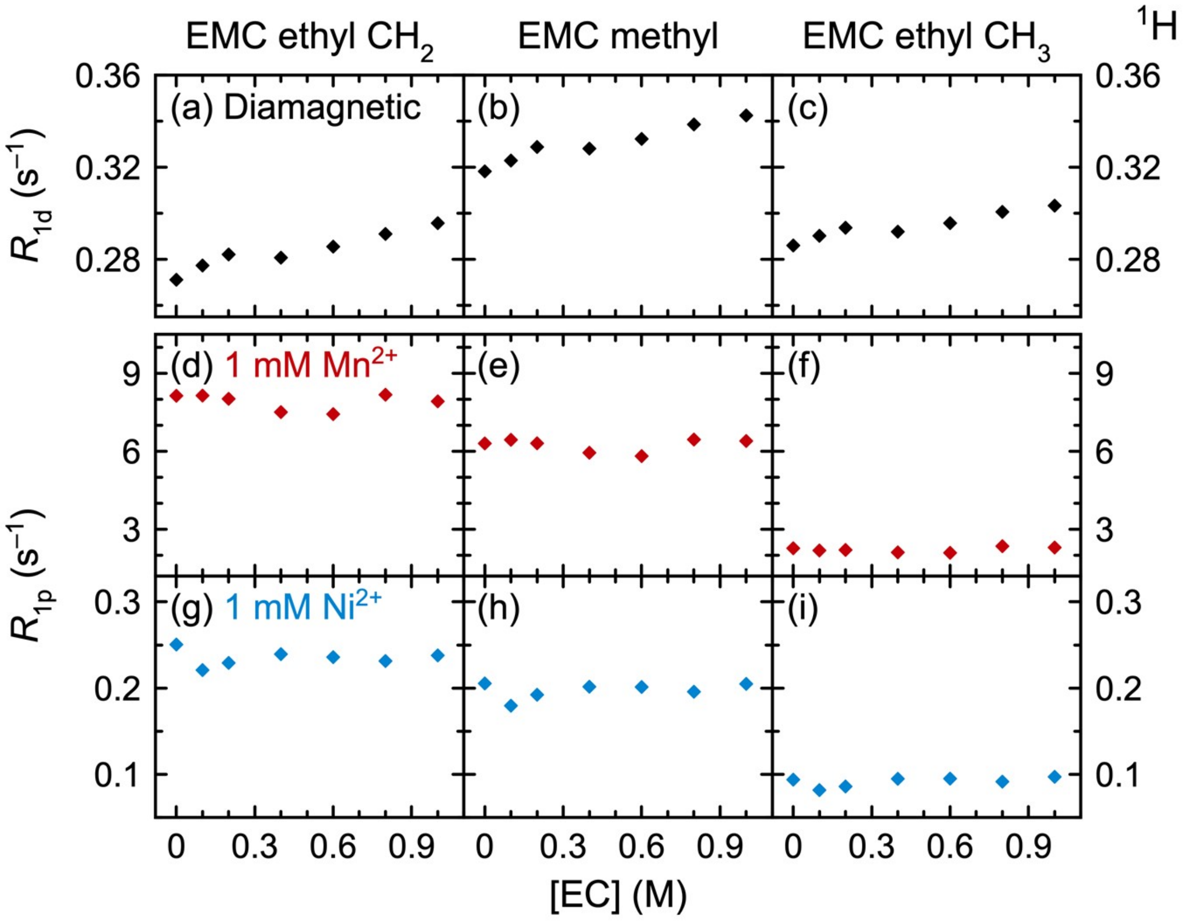

Figure S9. ^{1}H longitudinal nuclear relaxation rates of EMC in (a–c) diamagnetic, (d–f) Mn^{2+}-containing, and (g–i) Ni^{2+}-containing solutions of 1 M $LiPF_6$ in EMC with 0–1 M EC. R_{1d} (a–c) indicates the R_1 value of diamagnetic solutions, while R_{1p} (d–i) indicates the paramagnetic relaxation enhancement ($R_{1p} = R_1 - R_{1d}$). All measurements were performed at a field strength of 7.05 T.

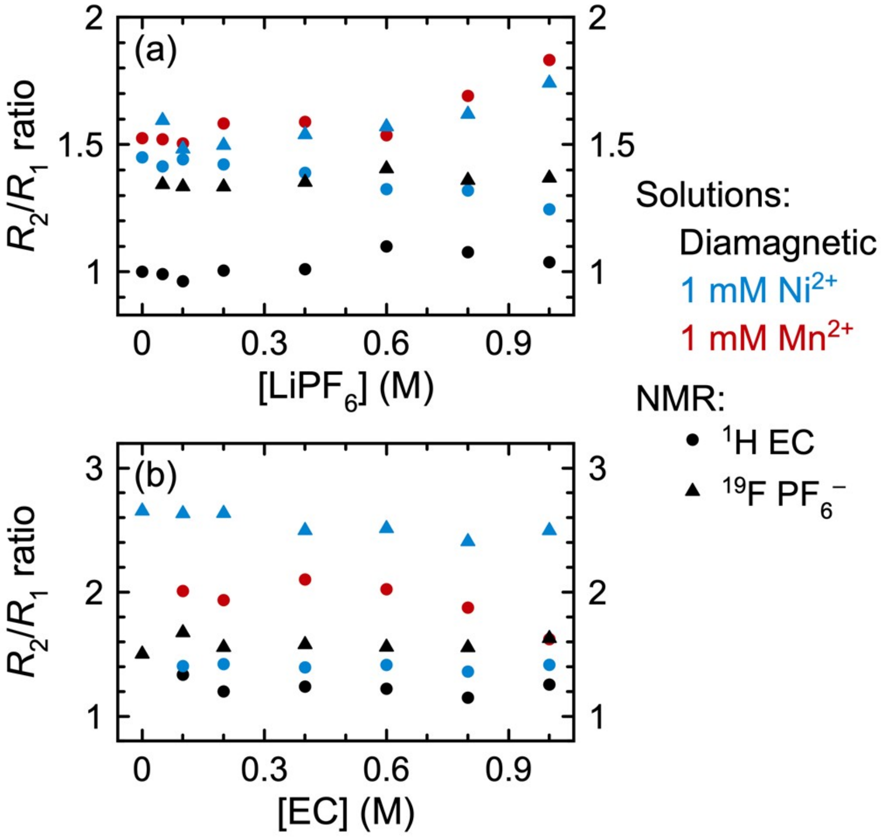

Figure S10. R_2/R_1 ratios of (a) solutions of 3:7 EC:EMC with 0–1 M $LiPF_6$ or (b) solutions of 1 M $LiPF_6$ in EMC with 0–1 M EC. Solutions are diamagnetic or contain Ni^{2+} or Mn^{2+}; relaxation of ^{1}H EC (circles) and ^{19}F PF_6^- (triangles) is shown. Full version including ^{19}F PF_6^- relaxation for Mn^{2+}-containing solutions is shown in Figure 5.

Figure S11 shows the R_2/R_1 ratios for the solutions whose R_{1p} values are shown in Figure 6. R_2/R_1 ratios are small for the diamagnetic solution (Figure S11a) and all the Ni^{2+}-containing solutions (Figure S11b). In the Mn^{2+}-containing solution (Figure S11c), the EC R_2/R_1 ratios are again smallest (1.80 average), followed by the $TFSI^-$ R_2/R_1 ratios (3.50 average), while the PF_6^- R_2/R_1 ratios are the largest by far (9.04 average). The R_2/R_1 ratios of these solutions are not inconsistent with the idea that Mn^{2+} prefers coordination to PF_6^- and Ni^{2+} prefers coordination to $TFSI^-$. In Mn^{2+}-containing solutions (Figure S11c), reducing the fraction of $TFSI^-$ in solution does not appear to lead to an increase in the ^{19}F PF_6^- R_2/R_1 ratio (unlike the increase that was observed in Figure 5b when the EC fraction was reduced). Although the ^{19}F $TFSI^-$ R_2/R_1 ratios are smaller than the ^{19}F PF_6^- R_2/R_1 ratios, this does not necessarily suggest that the PF_6^- residence time is longer than the $TFSI^-$ residence time, because different hyperfine constants apply, with small ^{19}F hyperfine coupling constants being predicted for $TFSI^-$ and thus smaller R_2/R_1 ratios.

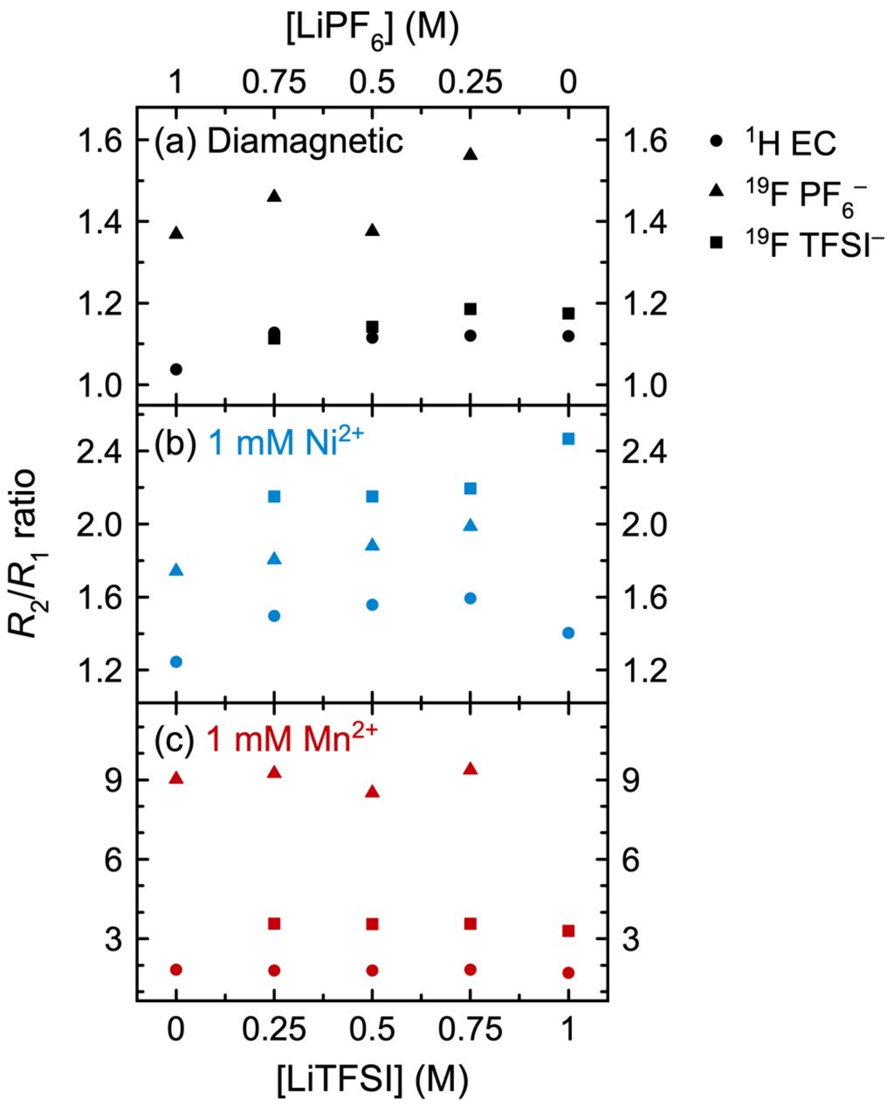

Figure S11. R_2/R_1 ratios of (a) diamagnetic, (b) Ni^{2+}-containing, or (c) Mn^{2+}-containing electrolyte solutions. Solutions comprised 3:7 EC:EMC (v/v) with 0–1 M $LiPF_6$ and 0–1 M LiTFSI, where the total Li^+ concentration remained constant at 1 M. Relaxation of ^1H EC (circles), ^{19}F PF_6^- (triangles), and ^{19}F $TFSI^-$ (squares) is shown.
